# Activated protein C promotes human lung cancer progression through the release of tumor extracellular vesicles and transfer of microRNA-200a

**DOI:** 10.1038/s41419-025-08173-z

**Published:** 2025-11-21

**Authors:** Madhura Chatterjee, Deepak Parashar, Rajan Pandey, Tanmoy Mukherjee, Saurabh Gupta, Subhojit Paul, Akash Chatterjee, Gunjan Potale, Prity Dhara, V. V. Sathibabu Uddandrao, S. Sengottuvelu, Aishwarya Sharma, Umesh Kumar, Jhansi Magisetty, Arindam Maitra, Kaushik Das

**Affiliations:** 1 Biotechnology Research and Innovation Council-National Institute of Biomedical Genomics, Kalyani, West Bengal India; 2 Regional Centre for Biotechnology, Ph.D. Program, Kalyani, India; 3 Division of Hematology & Oncology, Department of Medicine, Medical College of Wisconsin, Milwaukee, WI USA; 4 Translational Bioinformatics Group, International Centre for Genetic Engineering and Biotechnology, New Delhi, India; 5 Department of Physiology, University of Arizona, Tucson, AZ USA; 6 Clapp & Mayne Global Health Sector Consulting Group and Renaissance Information Systems (CAMRIS) International (under Contract No. 75N93019D00025 with National Institute of Allergy and Infectious Diseases), NIH, DHHS, Rockville, MD USA; 7 Department of Biotechnology, GLA University, Mathura, Uttar Pradesh India; 8 School of Biological Sciences, Indian Association for the Cultivation of Science, Kolkata, India; 9 Department of Biotechnology, Karpagam Academy of Higher Education (Deemed to be University), Coimbatore, Tamil Nadu India; 10 Centre for Active Pharmaceutical Ingredients, Karpagam Academy of Higher Education (Deemed to be University), Coimbatore, Tamil Nadu India; 11 Department of Pharmacology, Nandha College of Pharmacy, Erode, Tamil Nadu India; 12 Department of General Surgery, Sree Balaji Medical College and Hospital, Chromepet, Chennai India; 13 University Institute of Engineering, Chandigarh University, NH5, Gharuan, Chandigarh-Ludhiana Highway, Mohali, Punjab India; 14 Department of Zoology, Central University of Punjab, Bathinda, India

**Keywords:** Non-small-cell lung cancer, RNA

## Abstract

Blood coagulation and cancer are intricately related. Hypercoagulation associated with cancer leads to aberrant thrombin generation, which contributes to thrombosis. Thrombin also activates anticoagulant protein C and the activated protein C (aPC), in addition to regulating the coagulation pathway, it also elicits cell signaling by binding to endothelial cell protein C receptor (EPCR) and activating protease-activated receptor 1 (PAR1)-mediated cell signaling. Earlier studies showed that aPC promotes lung adenocarcinoma survival and metastasis. However, the underlying mechanism remains largely unknown. Our present study provides mechanistic insight into how aPC promotes lung adenocarcinoma survival, metastasis, and drug resistance. Our study shows that aPC, through EPCR-PAR1-driven activation of RhoA-ROCKII-JNK1/2-MLC2 signaling, triggers extracellular vesicle (EV) release from lung adenocarcinoma cells. aPC-EVs, via the transfer of microRNA (miR)-200a, promote proliferation, migration, and invasion of normal lung epithelial cells. They also confer resistance to lung cancer against chemotherapeutic agents. Inhibition of miR-200a functions through the incorporation of anti-miR-200a abrogates aPC-EVs-mediated tumorigenic effects. Furthermore, loading miR-200a mimic into control EVs showed similar phenotypic responses to that of aPC-EVs. miR-200a is shown to target SOX17 in the recipient cells, leading to tumorigenesis. miR-200a upregulation and SOX17 downregulation are consistently observed in lung cancer tissues in the UALCAN portal database of clinical specimens. Consistent with these findings, our in vivo studies in BALB/c nude mice showed that aPC-EVs from lung cancer cells promote tumor growth, metastasis, and drug resistance through miR-200a transfer. Targeting EV biogenesis, EV’s miR-200a, and/or EV uptake mechanisms may offer novel therapeutic strategies in limiting lung tumorigenesis, thereby increasing patients’ survival.

## Introduction

Blood coagulation and cancer are intrinsically connected [1, 2]; hypercoagulation-associated thrombotic complications such as venous thromboembolism (VTE) are commonly observed in certain cancer types such as lung cancer [3], accounting for increased morbidity and mortality of patients. The risk of VTE in lung cancer increases with cancer progression [4–6], in first line of treatment it varies from 5 to 13% [7–9]. Although hypercoagulation is well-established in lung cancer pathogenesis [3], how coagulation proteases influence lung cancer progression remains largely unknown. Thrombin is used as an emerging biomarker, assessing global activation of coagulation and increased risk of VTE in lung cancer patients [10]. Binding of thrombin with thrombomodulin triggers the activation of endothelial cell protein C receptor (EPCR)-bound protein C to generate activated protein C (aPC) [11]. aPC, in presence of co-factors [12], cleaves and inactivates activated factor V (FVa) and -VIII (FVIIIa) [13, 14], thereby serving as an anti-coagulant to restore homeostasis. Other than anti-coagulant functions, aPC-EPCR also promotes cytoprotective responses via the activation of protease-activated receptor 1 (PAR1) [15–17]. Interestingly, both EPCR [18] and PAR1 [19] are over-expressed in lung adenocarcinomas (LUAD), a subtype of non-small cell lung cancer (NSCLC), which are associated with poor prognosis and decreased patients’ survival [18, 19]. A higher expression of EPCR in the NSCLC tissues is correlated with lymph node metastasis, tumor size, and TNM stage [20]. Similarly, an up-regulation of PAR1 expression is also observed in NSCLC stroma as compared to normal lung tissues, indicating the possible diagnostic value of PAR1 in NSCLC pathogenesis [21]. aPC binding to EPCR promotes transcytosis of aPC from the lumen of blood vessels to the subendothelial tissues [22], thereby reaching the tumor cells [23]. Earlier evidence indicated that aPC-EPCR complex promotes the survival and metastasis of LUAD cells [18], however the role of PAR1 in this context remained unexplored. Additionally, the mechanistic details of how aPC influences LUAD progression remain ill-defined.

Data presented herein delineate that aPC-EPCR-mediated PAR1 activation induces release of extracellular vesicles (EVs) from A549 cells, the commonly used human LUAD cell line. We have demonstrated that RhoA-ROCKII-JNK1/2-MLC2 signaling axis plays a pivotal role in aPC-induced EV biogenesis. aPC triggers cellular miR-200a expression due to which EVs released upon aPC stimulation bear significant amount of miR-200a. Incorporation of aPC-released EVs but not control EVs not only promotes proliferation, migration, and invasion of normal lung epithelial cells, but also confers lung cancer resistance against chemotherapeutic drug-induced apoptosis. Inhibiting miR-200a significantly demolishes the effects of aPC-EVs. Moreover, enforced expression of miR-200a in control EVs shows similar phenotypic responses to that of aPC-EVs. We have demonstrated that aPC-EVs via miR-200a transfer elicit pro-tumorigenic effects by downregulating SOX17 expression and low SOX17 level is associated with poor survival of lung cancer patients. Consistent with these findings, our in vivo data also shows that incorporation of aPC-EVs but not control EVs into normal lung epithelial cells results in the formation of visible tumors in BALB/c nude mice. Perturbation of miR 200a not only reduces tumor volume and weight but also limits tumor metastasis. miR-200a-packaged control EVs again show similar phenotypes to that of aPC-EVs in vivo. Furthermore, when aPC-EVs were fused with naïve lung cancer cells, the EV-fused cells gained resistance against chemotherapeutic drug in vivo. These observations provide a new understanding of the role of aPC in lung tumorigenesis and offer developing potential therapeutics to limit the effect of aPC-EVs and associated lung cancer progression.

## Materials and methods

See Supplemental ‘Materials and Methods’ for additional details.

### Mice

All animal experiments were performed on 6-8-week-old BALB/c nude mice, obtained from Sri Lakshmi Narayana Institute of Medical Sciences & Hospital, Puducherry, India. The experiments were performed at Nandha College of Pharmacy, Erode, Tamil Nadu, India with Institutional Animal Ethics Committee approval from Sri Lakshmi Narayana Institute of Medical Sciences & Hospital, Puducherry, India (932/Po/Re/S/06/CPCSEA) for the proposal “Understanding the role of coagulation protease activated protein C (aPC) in the progression of lung cancer” (Proposal No. SLIMS/29/IAEC/2025-26). Both male- and female mice were equally distributed within each experimental group.

### Clinical data analysis

miR-200a expression in LUAD patient samples were analyzed using the UALCAN- (https://ualcan.path.uab.edu/) and Cancer MIRNome portal (http://bioinfo.jialab-ucr.org/CancerMIRNome/) [24]. SOX17 gene and protein expression in LUAD patient samples were analyzed using the UALCAN portal as previously mentioned (https://ualcan.path.uab.edu/*)* [25–27].

### Animal studies

For in vivo proliferation experiments, at first, A549 cells were treated with a control vehicle or aPC followed by the isolation of EVs. An equal number of control- and aPC-released EVs were incubated with BEAS-2B cells for 4 h. After washing, the EV-fused BEAS-2B cells (1 × 10^6^) were resuspended in 200 µL of serum-free media with Matrigel at 1:1 ratio and injected into the right flanks of BALB/c nude mice subcutaneously. Twenty-five days later, mice were euthanized, tumors were excised and imaged. Tumor size was measured in three dimensions, a, b, and c from which tumor volume was calculated using formula *abc* X 0.52 as mentioned earlier [28]. Tumor weight was also measured. The details of metastatic- and drug resistance model are included in ‘Supplemental Information’.

## Results

### aPC induces EV generation from lung cancer cells via EPCR-PAR1 signaling

**Fig. 1:**
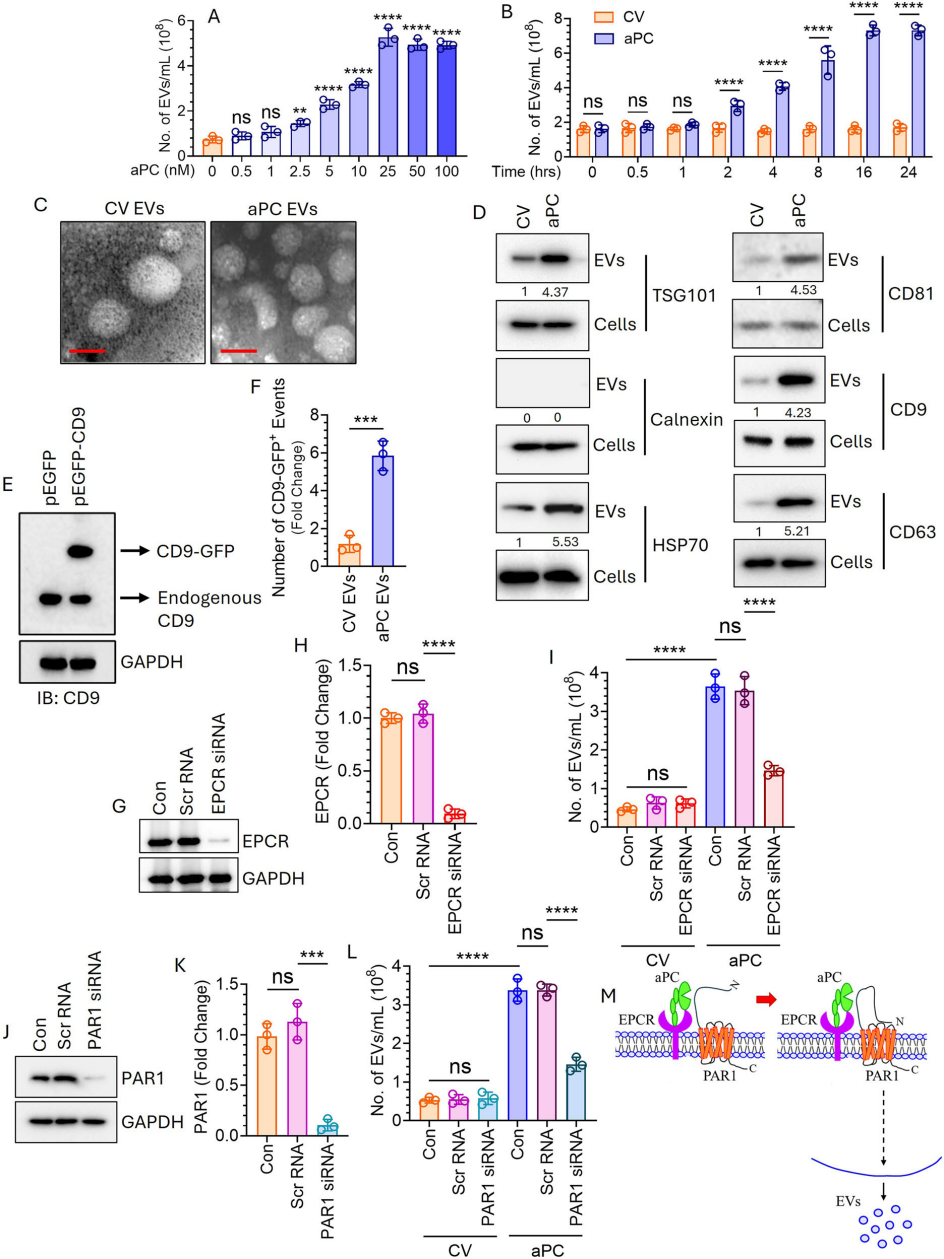
aPC promotes EV generation from human lung cancer cells via EPCR-dependent activation of PAR1. **A** A549 cells grown onto 6-well culture dishes (two wells together for each treatment) were serum starved for 1 h followed by exposure to varying concentrations of aPC (in CV, i.e., 20 mM Tris-HCl, pH 7.4, containing 100 mM NaCl;) for 16 h. EVs isolated from the supernatant were quantified by NTA Nano-Sight. **B** Cells were serum starved for 1 h followed by treatment with a control vehicle (CV; only 20 mM Tris-HCl, pH 7.4, containing 100 mM NaCl) or 25 nM of aPC for varying times. EVs were isolated from the supernatant and quantified by NTA Nano-Sight. **C** EVs derived from a CV or aPC were subjected to TEM analysis to determine the size and shape. Scale bar 200 nm. **D** Cells were treated with a CV or aPC for 16 h followed by the isolation of EVs. Both the EV- and cell lysates were subjected to immunoblot analysis to probe TSG101, Calnexin, HSP70, CD81, CD9, and CD63. **E** The cells were transfected by using lipofectamine 3000 with a plasmid reporter, pEGFP-N1, in which the EV marker, CD9 was cloned. After 48 h, the expression of CD9-GFP in the cells was analyzed by western blotting with CD9-specific antibody. **F** The transfected cells were serum starved for 1 h followed by treatment with a CV or aPC for 16 h. EVs were isolated from the culture supernatant and quantified by flow cytometry. **G** A549 cells were transfected with a scrambled RNA (Scr RNA; 200 nM) or EPCR-specific siRNA (EPCR siRNA; 200 nM) by using Lipofectamine 3000. Forty-eight hours later, the cells were lysed and EPCR expression was analyzed by western blotting with human EPCR-specific antibody. **H** Band intensities were quantified by densitometric analysis. **I** Scr RNA or EPCR siRNA-transfected cells were treated with a CV or aPC (25 nM) for 16 h. EVs isolated from the supernatant were quantified by NTA Nano-Sight. **J** Cells were transfected with a Scr RNA (200 nM) or PAR1-specific siRNA (200 nM) as mentioned in (**G**). The extent of PAR1 knockdown was analyzed by western blotting with human PAR1-specific antibody. **K** Band intensities were quantified by densitometric analysis. **L** Control- or PAR1 knocked down cells were challenged with a CV or aPC for 16 h. **M** Schematic diagram showing how EPCR-PAR1 signaling is involved in aPC-triggered EV generation from A549 cells. aPC binds EPCR and cleaves PAR1 at the N-terminal end. The newly generated N-termini act as a tethered ligand which bind PAR1 itself, leading to the induction of intracellular signaling, thereby triggering EV generation. EVs were isolated and quantified by NTA Nano-Sight. **P < 0.01; ***P < 0.001; ****P < 0.0001; ns not statistically significantly different.

The human LUAD cell line, A549, expressing both EPCR [18] and PAR1 [29], was exposed to varying concentrations of aPC and EV release was measured by Nano-Sight. The data indicated that aPC, in a concentration-dependent manner, markedly increased EV generation (Fig. 1A). Next, we treated cells with aPC (25 nM) for varying times and analyzed EV release. The data showed a time-dependent increase of EV generation by aPC (Fig. 1B). aPC-induced EVs appeared to be homogenous in size (~199 nm); in contrast, the control vehicle (CV)-treated EVs were highly heterogenous (~267 nm) (Supplementary Fig. 1A, B). Analysis of Exosomes (Exos), isolated by centrifugation of the EV conditioned media, demonstrated that aPC treatment did not influence the generation of Exos from lung cancer cells (Supplementary Fig. 2). We also characterized these EVs by TEM and observed spherical morphologies, typical EV-like structures in both CV- and aPC-treated group (Fig. 1C). The presence of transmembrane proteins (CD81, CD9, and CD63) associated with plasma membrane and cytosolic proteins (TSG101 and HSP70) recovered in EVs but not endoplasmic marker protein (Calnexin) in both CV- and aPC-EVs ensured successful purification of EVs (Fig. 1D). These proteins were enriched in aPC-treated group, reflecting the increase in EV generation (Fig. 1D). Next, we transfected the cells with a plasmid reporter, pEGFP-N1, in which the EV marker, CD9 was cloned (Fig. 1E). The transfected cells were treated with a CV or aPC. The EVs in the supernatant were isolated and quantified by flow cytometry, which indicates a marked increase in the CD9-GFP^+^ EVs upon aPC treatment (Fig. 1F). Further, we tested the effect of aPC on EV generation in another human lung cancer cell line, HCC827. Treatment of HCC827 with aPC also resulted in enhanced release of EVs (Supplementary Fig. 3). To understand the role of aPC-EPCR-PAR1 signaling in EV generation, first, EPCR was silenced in A549 cells using specific siRNA (Fig. 1G, H) and EV release was analyzed following treatment with a CV or aPC which showed a significant downregulation in aPC-mediated EV generation upon EPCR knockdown (Fig. 1I). Next, we knocked down PAR1 in the cells (Fig. 1J, K) followed by treatment with a CV or aPC and EV release was monitored. PAR1 knockdown showed a significant inhibition of EV generation by aPC (Fig. 1L). Taken together, data in Fig. 1 delineates that aPC triggers EV generation from human lung cancer cells through EPCR-PAR1 (Fig. 1M).

### aPC triggers EV generation via RhoA-ROCKII-JNK1/2-MLC2 pathway

**Fig. 2:**
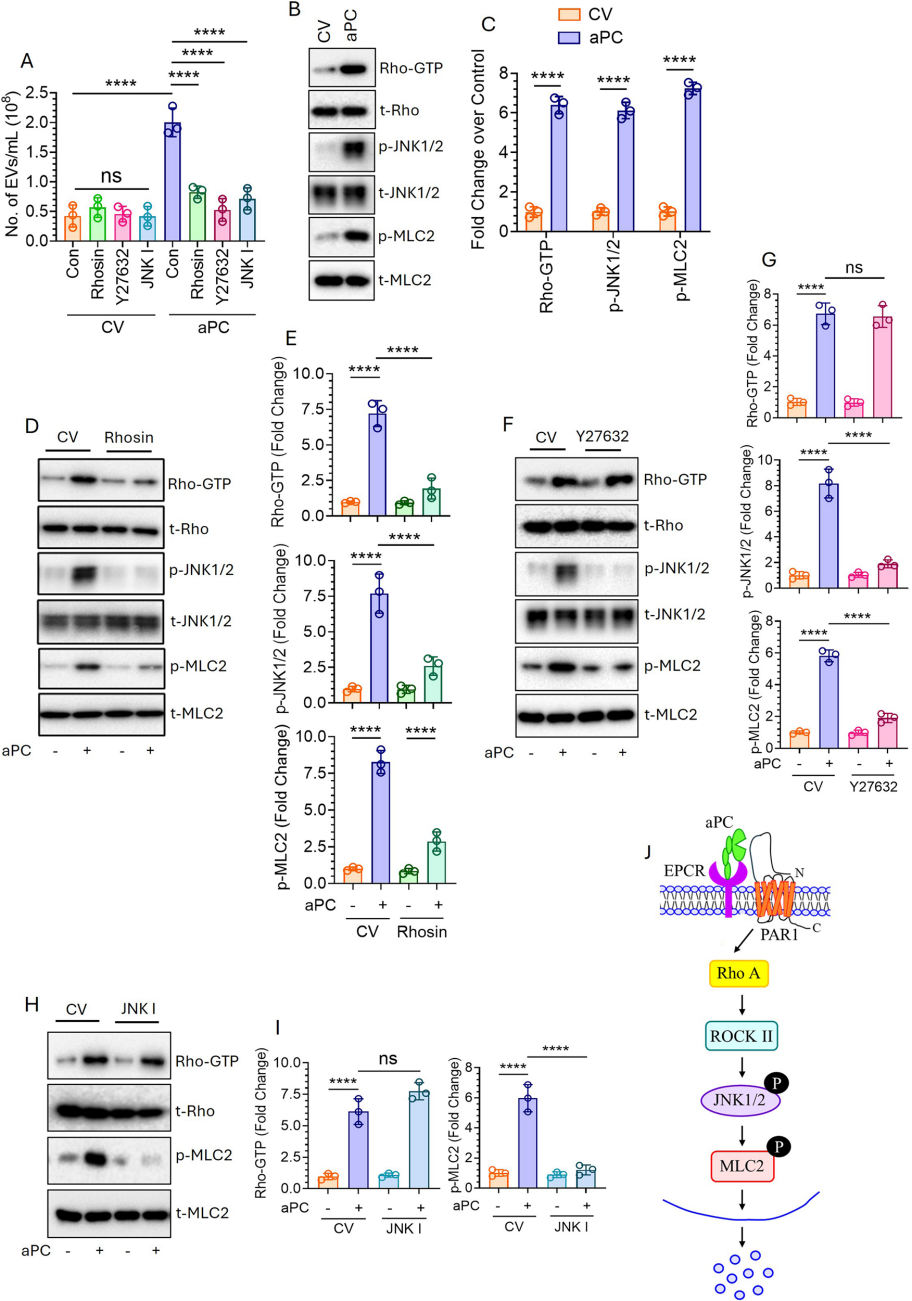
aPC-triggered EV generation from human lung cancer cells involves intracellular activation of the RhoA-ROCKII-JNK1/2-MLC2 pathway. **A** A549 cells were serum starved for 1 h followed by the pre-treatment of Rhosin (50 µM) or Y27632 (10 µM) or JNK I (SP600125; 20 µM) for another 1 h. Cells were then challenged with a CV or aPC (25 nM) for 16 h. EVs in the supernatant were quantified by NTA Nano-Sight. **B** Cells were starved for 1 h followed by treatment with a CV or aPC (25 nM). Cells were lysed after 10 min, 1 h, and 2 h of treatment to analyze the level of RhoA-GTP level and phosphorylation of JNK1/2, and MLC2, respectively by western blotting. RhoA activation was measured by analyzing the GTP-bound RhoA using the RhoA activation assay kit. **C** Band intensities were quantified by densitometric analysis. **D** Cells were pre-treated with Rhosin for 1 h followed by CV or aPC challenge. RhoA activation and phosphorylation of JNK1/2 and MLC2 were measured. **E** Band intensities were quantified after performing the densitometric analysis. **F** Cells were serum-starved for 1 h followed by pre-treatment with a CV or Y27632 for another 1 h. After the indicated time, cells were challenged with a CV or aPC and RhoA, JNK1/2, and MLC2 activation was analyzed after 10 min, 1 h, and 2 h, respectively. **G** Band intensities were quantified after densitometric analysis. **H** Serum-starved cells were treated with a CV or JNK I for 1 h, followed by the exposure of a CV or aPC. After 10 min and 2 h, RhoA activation and MLC2 phosphorylation were analyzed and **I** accordingly densitometric analysis was performed to quantify band intensities. **J** Schematic representation demonstrates that aPC binding to EPCR triggers PAR1 signaling to induce RhoA activation. The RhoA signaling promotes ROCKII activation which further promotes JNK1/2 phosphorylation. Phospho-JNK1/2 further promotes MLC2 phosphorylation, which triggers the release of EVs. ***P < 0.001; ****P < 0.0001; ns not statistically significantly different.

MLC2 plays a key role in EV biogenesis via modulating myosin activity [30, 31]. MLC2 phosphorylation is often regulated by JNK1/2 [32], which is activated by ROCKII [33]. ROCKII activation is further triggered by RhoA [34]. To investigate the role of RhoA-ROCKII-JNK1/2-MLC2 in EV biogenesis, A549 cells were pre-treated with potent inhibitors of RhoA, ROCKII and JNK1/2; Rhosin, Y27632 and JNK I, respectively followed by aPC challenge and EV generation was measured. Perturbation of RhoA or ROCKII or JNK1/2 significantly downregulated aPC-release of EVs (Fig. 2A). Activation of JNK1/2, and MLC2 in cells were determined by measuring their phosphorylation status whereas RhoA activation was measured by analyzing the GTP-bound state. aPC treatment showed a significant activation of all three molecules (Fig. 2B, C). Next, we determined the chronology of activation of all these signaling molecules. First, RhoA-inhibited cells were tested for RhoA-GTP level as well as JNK1/2 and MLC2 phosphorylation. RhoA inhibition significantly downregulated aPC-induced RhoA activation and phosphorylation of JNK1/2 and MLC2 (Fig. 2D, E), indicating that RhoA lies upstream of JNK1/2 and MLC2. Next, we inhibited ROCKII and analyzed RhoA activation as well as JNK1/2, and MLC2 phosphorylation. The perturbation of ROCKII did not interfere with RhoA activation, rather downregulated both JNK1/2 and MLC2 phosphorylation (Fig. 2F, G), signifying that ROCKII lies downstream of RhoA but upstream of JNK1/2 and MLC2. Finally, JNK1/2 inhibited cells were analyzed for RhoA and MLC2 activation. JNK1/2 inhibition did not interfere with RhoA activation but completely attenuated MLC2 phosphorylation (Fig. 2H, I), demonstrating JNK1/2 as downstream of RhoA but upstream of MLC2. In summary, data in Fig. 2 reflects that aPC-driven activation of RhoA-ROCKII triggers phosphorylation of JNK1/2. Activated JNK1/2 further phosphorylates MLC2 which promotes EV generation (Fig. 2J).

### aPC-EVs from lung cancer promote proliferation, migration, invasion, and chemoresistance

**Fig. 3:**
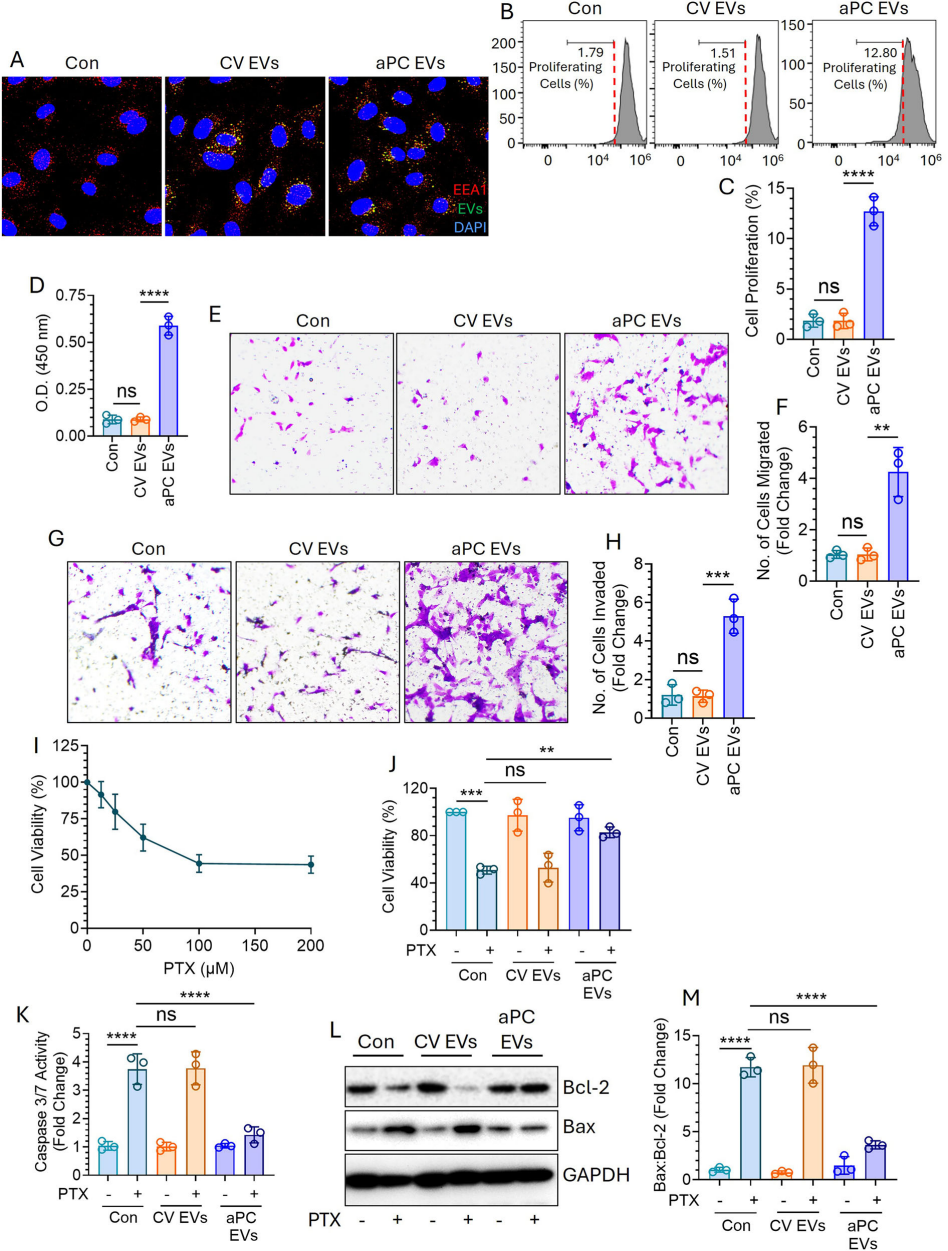
aPC-derived EVs from A549 cells promote proliferation, migration, and invasion of BEAS-2B cells while conferring A549 cells resistance against PTX. **A** A549 cells were labeled with fluorescent PKH67 dye (green) followed by the treatment of a CV or aPC. After 16 h, EVs were isolated from the cell supernatant and an equal number of EVs (1 ×10^8^) were fused with BEAS-2B cells (cells:EVs = 1:100) for 4 h. After washing twice to remove the unbound EVs, the cells were fixed and immuno-stained with anti-EEA1 antibody (red) to stain the early endosomes. The appearance of yellow confirms the uptake of EVs via endocytosis. DAPI was used to stain the nuclei (blue). **B** BEAS-2B cells grown onto 6-well culture dishes (two wells together for each treatment) were treated with carboxyfluorescein diacetate succinimidyl ester (CFSE; 10 µM) for 20 min at 37 °C. After washing twice with 1X HBSS, the cells were incubated with a CV or (Con) or equal number (1 × 10^8^) of CV-EVs or aPC-EVs from A549 cells for 4 h. After washing to remove the unbound EVs, cells were incubated for another 20 h at 37 °C and then subjected to analysis of proliferation by flow cytometry and **C** percentage of cell proliferation was calculated. **D** BEAS-2B cells were fused with equal number (1 × 10^8^) of CV-EVs or aPC-EVs from A549 cells followed by culturing in a medium containing 5-bromo-2-deoxyuridine (BrdU; 10 µM) for 8 h. Cells were then fixed with paraformaldehyde, washed with HBSS, and permeabilized with 0.01% Triton X-100. After blocking with 5% BSA, the cells were incubated overnight with anti-BrdU antibody at 4 °C. After washing, probing with HRP-conjugated secondary antibody was done for 1 h followed by the addition of tetramethylbenzidine substrate with H_2_O_2_. O.D. was measured at 450 nm to determine the degree of cell proliferation. **E** EV-fused BEAS-2B cells were placed in serum-free media on top of a trans-well membrane (0.8 µm pore size) while the bottom compartment was filled with FBS-containing media. After incubation at 37 °C for 24 h, cells on the upper surface of the membrane were scraped off and the migrated cells through the pores in the lower surface of the membrane were stained with crystal violet (CV) solution (0.1% CV, 0.1 M borate, and 2% ethanol). Images were taken using a bright-field microscope from which **F** the number of cells migrated was quantified. **G** EV-fused cells in a serum-free media were placed on top of a trans-well membrane which was previously coated with Matrigel. The lower compartment was filled with FBS-containing media. After incubation at 37 °C for 48 h, the cells on upper surface of the membrane were removed whereas the invaded cells through the Matrigel in lower surface were stained with CV solution and imaged. **H** From the images, the number of invaded cells was quantified. **I** A549 cells grown in 96-well culture plates were treated with different concentrations of PTX (0, 12.5, 25, 50, 100, and 200 µM) for 48 h. The media was then replaced, and the cells were incubated in 200 μL of fresh media containing 0.5 mg/mL 3-(4,5-dimethylthiazol-2-yl)-2,5-diphenyltetrazolium bromide (MTT) at 37 °C for 4 h. The supernatant was removed and 200 µL of DMSO was added to lyse the cells for 10 min at 37 °C. The O.D. was measured at 570 nm from which % cell viability was calculated. **J** An equal number (1 × 10^8^) of CV-EVs or aPC-EVs from A549 cells was incubated with naïve A549 cells for 4 h. Both the control- and EV-fused cells were treated with PTX (100 µM) for 48 h. Cell viability was determined by MTT assay as described in (**I**). **K** The control- and EV-fused (CV-EVs and aPC-EVs) A549 cells were treated with PTX for 24 h followed by analyzing Caspase 3/7 activity as mentioned in ‘Materials and Methods’. **L** Both the control- and EV-fused (CV-EVs and aPC-EVs) A549 cells were exposed to PTX for 24 h. The expression of Bcl-2 and Bax was analyzed by western blotting. **M** Bax/Bcl-2 ratio was determined after measuring band intensities by densitometric analysis. **P < 0.01; ***P < 0.001; ****P < 0.0001; ns not statistically significantly different.

We incubated an equal number of PKH67-labeled CV- or aPC-EVs from A549 cells with normal lung epithelial cells, BEAS-2B. Both CV- and aPC-EVs were taken up by BEAS-2B with equal efficiency (Fig. 3A). EV-fused BEAS-2B cells were subjected to proliferation analysis by CFSE cell proliferation assay (Fig. 3B, C) and BrdU incorporation assay (Fig. 3D). Unlike control or CV-EVs, aPC-EVs promoted BEAS-2B proliferation significantly. Additionally, aPC-EV-fused BEAS-2B showed a higher degree of migration through trans-well inserts (Fig. 3E, F) and invasion through Matrigel-paved trans-well inserts (Fig. 3G, H). Further, these EVs were tested for conferring chemotherapeutic resistance. The treatment of paclitaxel (PTX) to A549 cells resulted in the loss of cell viability in a dose-dependent manner (Fig. 3I). However, PTX-induced cell death was significantly attenuated upon incorporation of aPC-EVs; equal number of CV-EVs did not show any effect (Fig. 3J). PTX is known for inducing lung cancer apoptosis [35], hence we tested the effect of these EVs in modulating PTX-induced A549 apoptosis by measuring caspase 3/7 activity and Bax:Bcl-2 level. PTX-induced increase in caspase 3/7 activity or Bax:Bcl-2 level was reduced upon incorporation of aPC-EVs (Fig. 3K–M).

### EV-mediated transfer of miR-200a promotes lung tumorigenesis in vitro

**Fig. 4:**
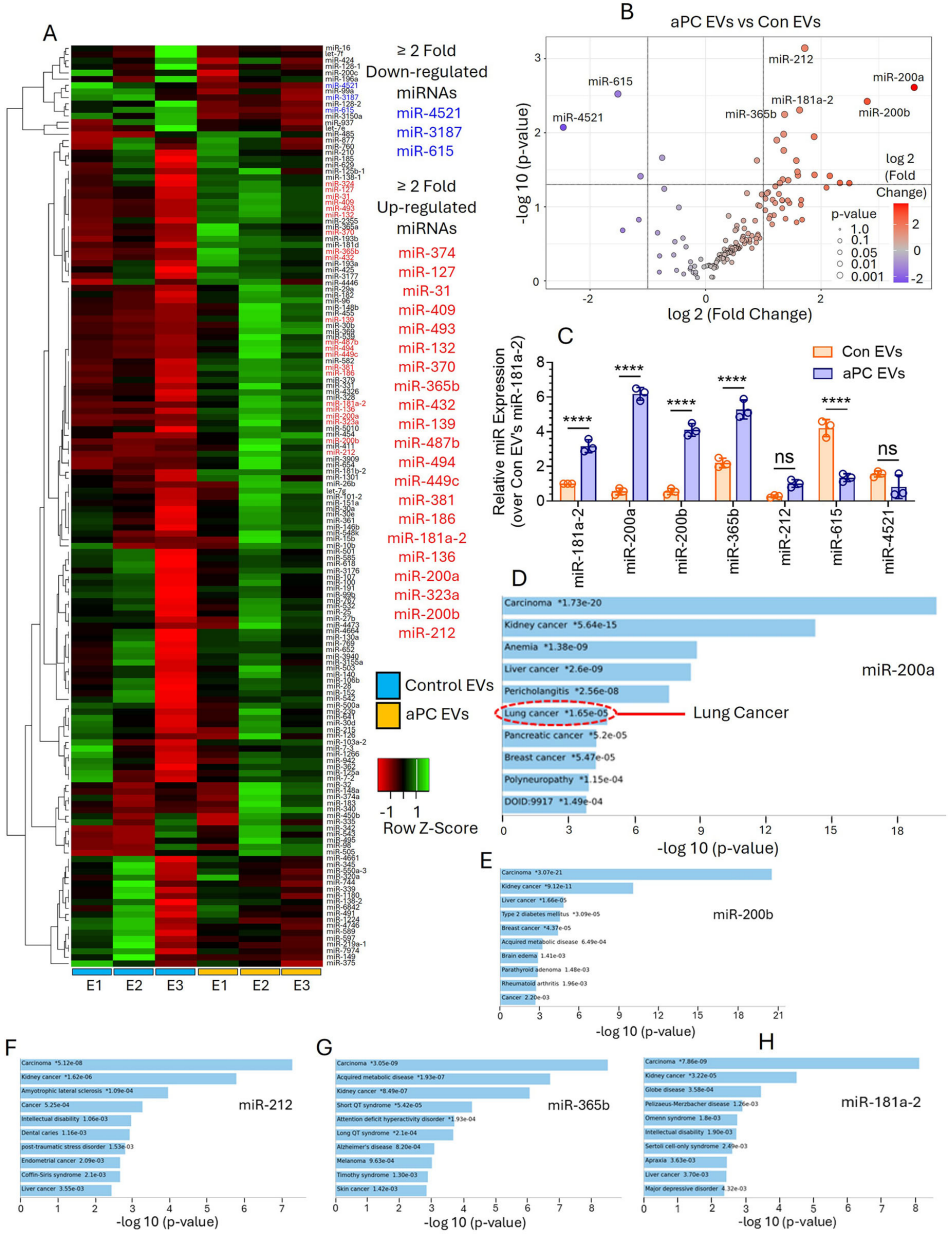
miRNA profiling in control (CV)- versus aPC-EVs relating to the pathogenesis of lung cancer. **A** EVs isolated from A549 cells after treatment with a CV or aPC (25 nM) were quantified by NTA Nano-Sight. An equal number of EVs (1 × 10^9^) were subjected to miR analysis by deep sequencing (Novogene Corporation Inc., Durham, North Carolina, USA). From the sequencing data, the top 150 most abundant and differentially expressed (DE) miRs were shown in the heat map. **B** The DE miRs between the aPC-derived EVs and con EVs with the fold change of >2.0 and adjusted *p*-value of <0.05 were shown in the volcano plot. **C** Validation of the DE miRs in control EVs and aPC-released EVs from the sequencing data. A549 cells were challenged with a CV (Con) or aPC (25 nM) for 16 h. The EVs released in the supernatant were subjected to isolation of miRs by mirPremier microRNA Isolation Kit. The relative abundance of miR-181a-2, miR-200a, miR-200b, miR-365b, miR-212, miR-615, and miR-4521 in equal number (1 ×10^8^) of Con EVs versus aPC-EVs was analyzed by real time PCR. Network analysis to explore the connections between different diseases and **D** miR-200a, **E** miR-200a, **F** miR-212, **G** miR-365b, and **H** miR-181a-2 were performed by using Jensen Diseases Database. *P < 0.05; ***P < 0.001; ****P < 0.0001.

An equal number of CV- or aPC-EVs from A549 cells were analyzed for microRNA (miR) by deep sequencing (Novogene Corporation Inc., Durham, NC, USA). Data indicated that 582 different miRs were present in both EV types with an abundance from 278886 to 0.33 transcripts, out of which 414 were present in >10 transcripts. First 150 differentially expressed (DE) miRs were shown in Fig. 4A. Among these DE miRs, 21 were significantly upregulated by ≥2-fold and 3 were significantly downregulated by ≥2-fold (Fig. 4B). Among the upregulated DE miRs, miR-200a, miR-200b, miR-212, miR-181a-2, and miR-365b whereas among the downregulated ones, miR-4521 and miR-615 showed higher statistical significance (Fig. 4B). Next, we validated the expression of the above-mentioned miRs in the EVs by qRT-PCR. Consistent to our sequencing data, we observed a significant increase of miR-200a, miR-200b, miR-212, miR-181a-2, and miR-365b level whereas downregulation of miR-4521 and miR-615 in aPC-EVs as compared to CV-EVs (Fig. 4C). miR-200a showed the highest increase (~7-fold) (Fig. 4C). To investigate functional implications of the identified upregulated miRs, we performed a network analysis to explore the connections between miRs and various diseases using the Jensen Diseases database (Fig. 4D–H) [36]. Data revealed that all upregulated miRs in aPC-EVs showed significant connections with different types of cancer and other diseases; however, only miR-200a were found to be associated with lung cancer (Fig. 4D).

**Fig. 5:**
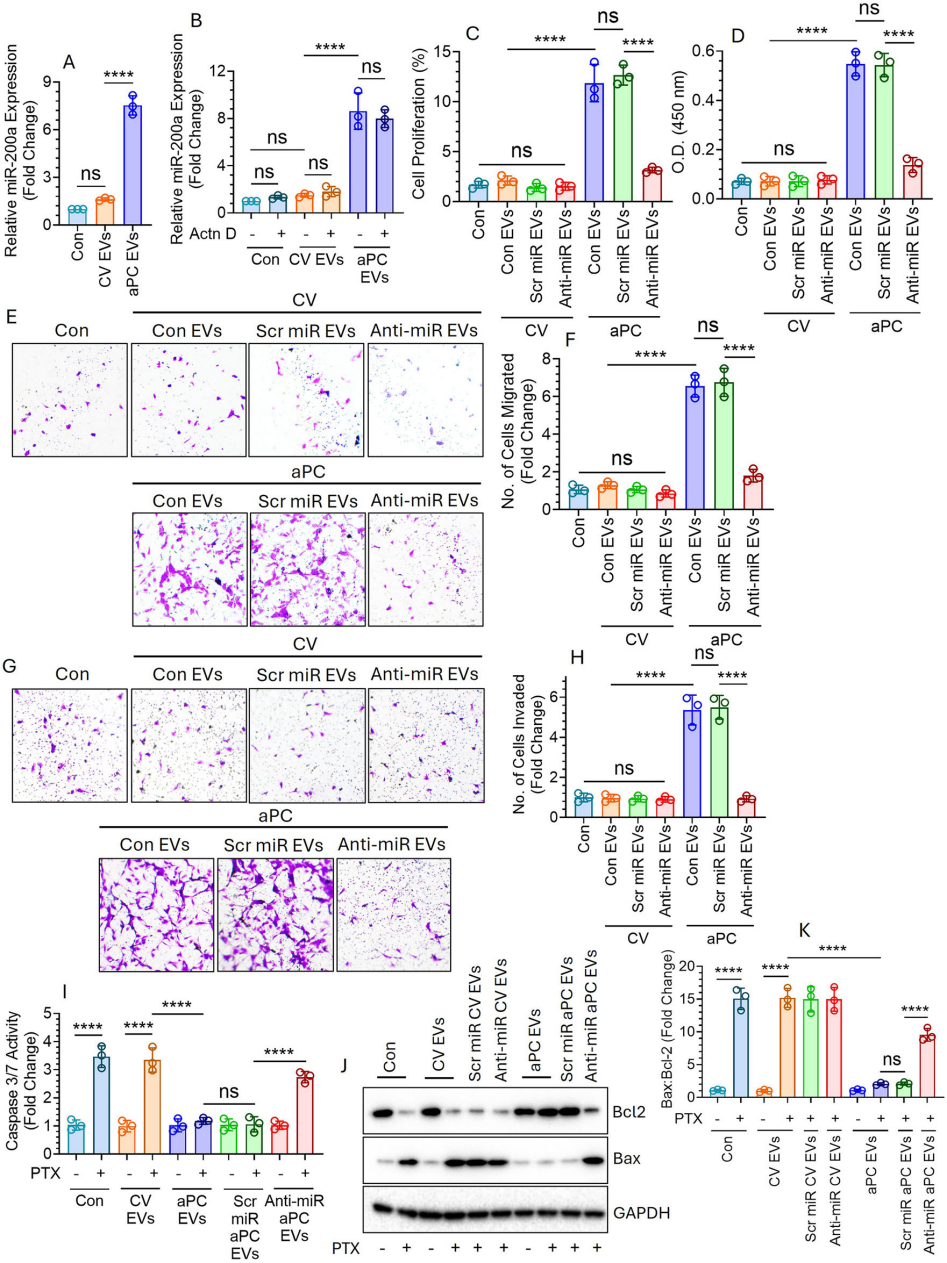
aPC-EV-mediated transfer of miR-200a promotes proliferation, migration, and invasion of BEAS-2B cells while conferring A549 cells resistance against PTX. **A** EVs isolated from A549 cells after treatment with a CV or aPC were incubated with BEAS-2B cells in equal number (1 × 10^8^) for 4 h. After washing twice with HBSS to remove the free EVs, miR-200a expression in the recipient BEAS-2B cells was analyzed by real time PCR. **B** BEAS-2B cells were pre-treated with actinomycin D (Actn D; 10 µg/mL) for 8 h followed by the fusion of the EVs. miR-200a expression in the EV-fused recipient BEAS-2B cells were analyzed by real time PCR. **C**, **D** A549 cells were transfected with 20 nM of scrambled miR (Scr miR) or anti-miR 200a (Anti-miR) followed by the exposure of a CV or aPC. The EVs so generated were fused with BEAS-2B cells in equal number (1 × 10^8^) and cell proliferation was analyzed by **C** CFSE proliferation assay and **D** BrdU proliferation assay. **E**–**H** Scr miR or Anti-miR-transfected A549 cells were treated with a CV or aPC followed by the isolation of EVs. The EVs were fused with BEAS-2B cells in equal number (1 × 10^8^) and EV-fused recipient BEAS-2B (**E**, **F**) migration and (**G**, **H**) invasion was determined. **I–K** EVs isolated from A549 cells, which were transfected with Scr miR or Anti-miR followed by CV- or aPC challenge, were fused with naïve A549 cells in equal number (1 × 10^8^). EV-fused A549 cells were exposed to PTX followed by analysis of **I** caspase activity and **J**, **K** Bax/Bcl-2 ratio as a measure of apoptosis. ****P < 0.0001; ns not statistically significantly different.

We fused an equal number of CV- or aPC-EVs from A549 cells with BEAS-2B and analyzed miR-200a expression in EV-fused BEAS-2B cells (Fig. 5A). Incorporation of aPC-EVs but not CV-EVs significantly enhanced miR-200a level in BEAS-2B cells. Pre-treatment of cells with Actinomycin D (Actn D) did not interfere with the enhanced expression of miR-200a in aPC-EV-fused BEAS-2B cells, indicating aPC-EV-mediated delivery of miR-200a rather than the de novo synthesis of miR-200a in the recipient cells (Fig. 5B). Actn D pre-treatment significantly reduced the expression of a transient gene, *c-myc* in BEAS-2B cells, demonstrating the effective transcriptional inhibition by Actn D (Supplementary Fig. 4A). The increased expression of miR-200a in aPC-EVs may stem from enhanced miR-200a expression in the cells as aPC treatment induced miR-200a expression in A549 cells (Supplementary Fig. 4B). Next, to investigate the role of miR-200a in aPC-EV-induced lung cancer progression, we transfected A549 cells with a scrambled miR (Scr) or miR-200a inhibitor (anti-miR) followed by treatment of a CV or aPC. The released EVs were fused with BEAS-2B to analyze cell proliferation (Fig. 5C, D), migration (Fig. 5E, F), and invasion (Fig. 5G, H). The data indicated that aPC-EV-induced cell proliferation, migration and invasion were reversed upon introduction of anti-miR-200a. Treatment of anti-miR-200a not only reduced miR-200a level in the EVs (Supplementary Fig. 4C) but also downregulated miR-200a in EV-fused recipient cells (Supplementary Fig. 4D). These EVs were also fused with naïve A549 cells to analyze PTX-induced cell death (Fig. 5I–K). PTX-triggered cell death was significantly reduced upon incorporation of aPC-EVs, however, anti-miR-200a again reversed the effect of aPC-EVs, delineating the importance of miR-200a in drug resistance (Fig. 5I–K). Inhibition of other upregulated miRs (miR-200b, miR-212, miR-181a-2, and miR-365b) in aPC-EVs did not interfere with enhanced proliferation, migration, invasion, and drug resistance (Supplementary Fig. 5A–D). Hence, we focused on understanding the role of miR-200a in aPC-EV-driven pathogenesis of lung cancer.

Next, A549 cells were transfected with either a Scr miR or miR-200a mimic followed by treatment of only the CV (no aPC was used). CV-EV-fused recipient cells were subjected to analysis of cell proliferation, migration, invasion, and response against PTX. The introduction of miR-200a mimic not only enhanced miR-200a level in the mimic EVs (Supplementary Fig. 6A) but also increased miR-200a expression in mimic EV-fused recipient cells (Supplementary Fig. 6B). Interestingly, mimic EV-fused recipient cells underwent enhanced proliferation (Supplementary Fig. 6C, D), migration (Supplementary Fig. 6E, F), invasion (Supplementary Fig. 6G, H), and PTX resistance (Supplementary Fig. 6I–K).

### aPC-EVs promote lung tumorigenesis via miR-200a-dependent SOX17 downregulation

**Fig. 6:**
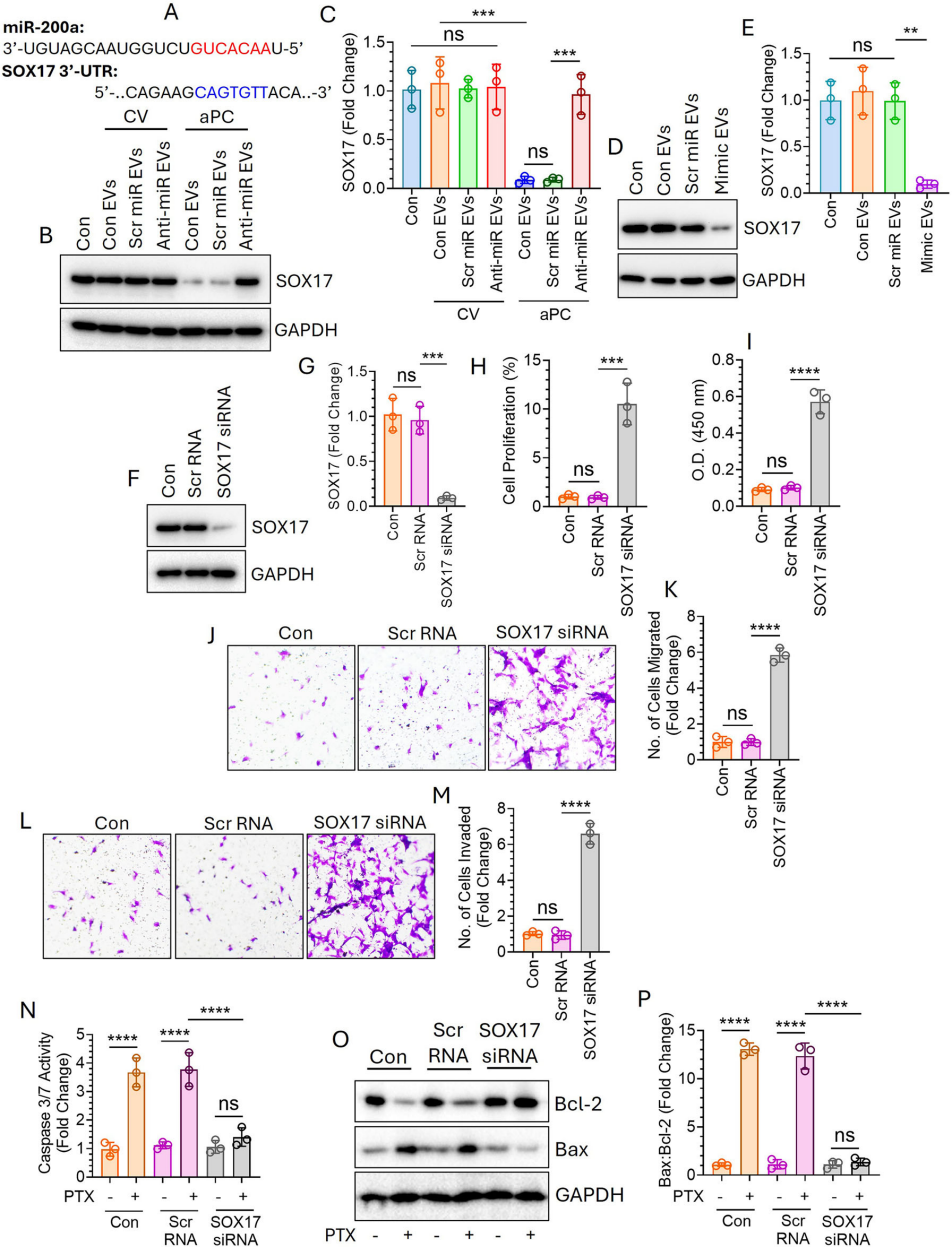
EV-mediated delivery of miR-200a promotes cell proliferation, migration, and invasion as well as confers drug resistance through the downregulation of SOX17. **A** miR-200a binding site within the 3’-UTR of SOX17. **B** A549 cells were transfected with Scr miR or Anti-miR followed by the treatment of a CV or aPC for 16 h. EVs were isolated from the culture supernatant and incubated with BEAS-2B cells in equal number (1 × 10^8^). After 24 h, the expression of SOX17 in the EV-fused recipient cells was analyzed by western blotting and **C** accordingly band intensities were quantified by densitometric analysis. **D** EVs were also isolated from the supernatant of Scr miR or miR Mimic-transfected A549 cells and fused in equal number (1 × 10^8^) with BEAS-2B cells. After 24 h, the expression of SOX17 in EV-fused BEAS-2B cells was analyzed by western blotting. **E** Band intensities were quantified by densitometric analysis. **F** BEAS-2B cells were transfected with Scr RNA or SOX17-specific siRNA. After 48 h, the cells were lysed and expression of SOX17 was analyzed by western blotting. **G** Band intensities were quantified by densitometric analysis. SOX17 knocked down BEAS-2B cells were analyzed for cell proliferation by **H** CFSE proliferation assay or **I** BrdU incorporation assay, **J**, **K** migration, and **L**, **M** invasion. SOX17 was also silenced in A549 cells followed by treatment with PTX for 48 h. The extent of apoptosis was measured by analyzing **N** Caspase 3/7 activity and **O**, **P** Bax/Bcl-2 ratio. **P < 0.01; ***P < 0.001; ****P < 0.0001; ns not statistically significantly different.

miR-200a has a putative binding site in the 3’-UTR of SOX17 (Fig. 6A), a well-known target of miR-200a [37–39]. We analyzed the expression of SOX17 in EV-fused recipient cells (Fig. 6B–E). The data clearly suggested that the incorporation of aPC-EVs significantly downregulated SOX17 expression in recipient cells which was completely restored upon introduction of anti-miR-200a (Fig. 6B, C). Like aPC-EVs, miR-200a mimic-loaded control EVs also showed significant downregulation of SOX17 expression in the recipient cells (Fig. 6D, E). Next, we knocked down SOX17 in BEAS-2B cells (Fig. 6F, G) and analyzed cell proliferation (Fig. 6H, I), migration (Fig. 6J, K), and invasion (Fig. 6L, M). SOX17-silenced cells showed enhanced cell proliferation, migration, and invasion. Moreover, A549 cells became resistant to PTX after silencing SOX17 (Fig. 6N–P).

### High miR-200a and low SOX17 expression in patients with LUAD is associated with poor prognosis and decreased patients’ survival

**Fig. 7:**
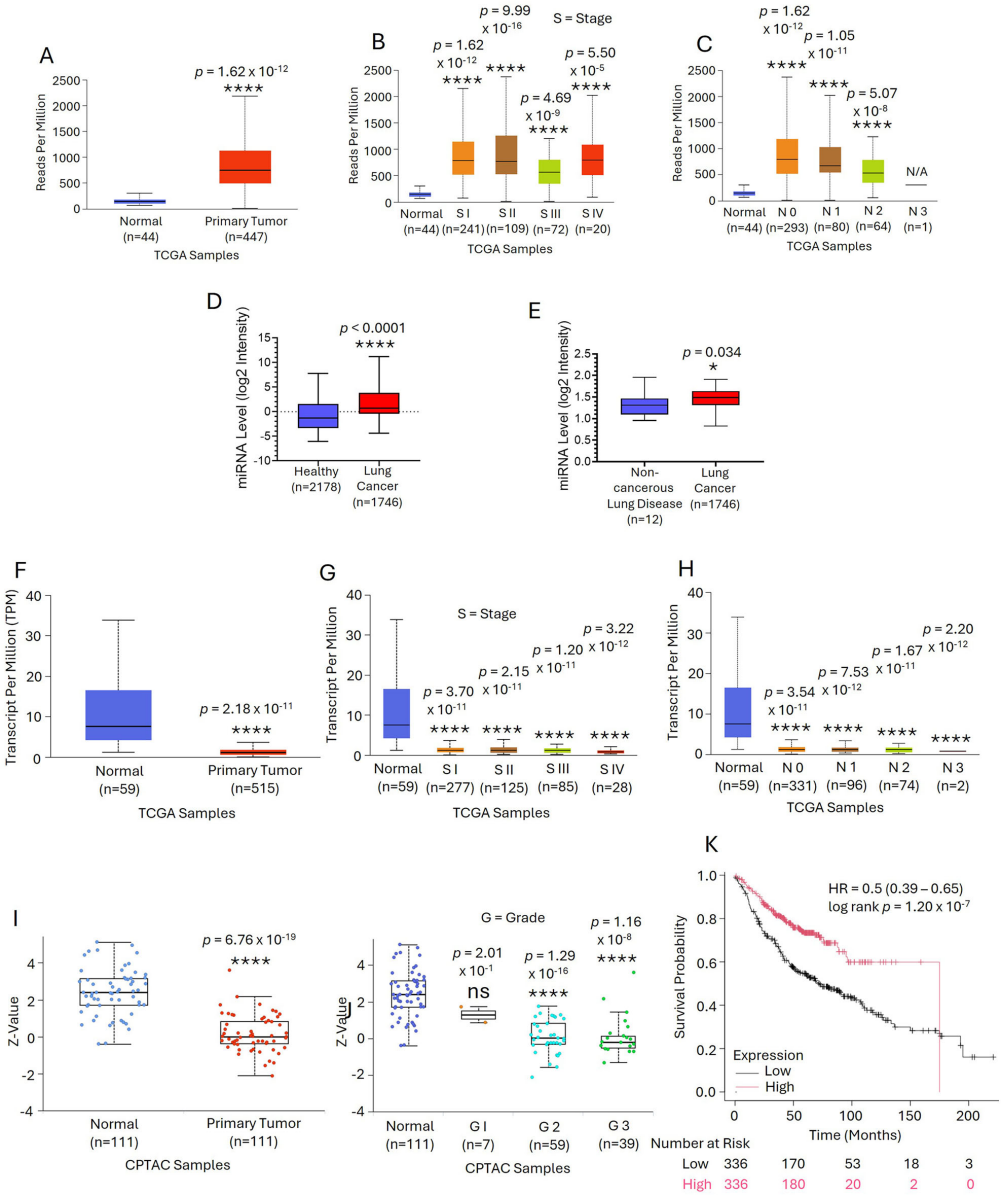
Up-regulation of miR-200a and down-regulation of miR-200a target, SOX17 was observed in LUAD patients as compared to healthy control. **A** miR-200a expression in primary LUAD tumors (*n* = 447) with respect to normal lung tissues (*n* = 44) as identified from TCGA database. **B** miR-200a expression across different LUAD stages as compared to normal (*n* = 44). **C** miR-200a level in different LUAD nodal metastasis statuses relative to normal samples (*n* = 44). Pathologic ‘N’ descriptions: N0, No regional lymph node metastasis; N1, Metastases in 1–3 axillary lymph nodes; N2, Metastases in 4–9 axillary lymph nodes; N3, Metastases ≥10 axillary lymph nodes. **D** miR-200a expression in lung cancer patients’ sera as compared to healthy controls (GSE137140). **E** miR-200a expression in lung cancer patients’ sera as compared to non-cancerous lung diseases (GSE68951). **F** SOX17 gene expression in primary LUAD tumors (*n* = 515) compared to normal lung tissues (*n* = 59). **G**, **H** SOX17 gene expression across different LUAD stages and nodal metastasis statuses relative to normal samples (*n* = 59). **I** SOX17 protein expression in primary LUAD tumors (*n* = 111) compared to normal lung tissues (*n* = 111). **J** SOX17 protein expression across different LUAD tumor grades. **K** Kaplan–Meier survival analysis of LUAD patients, stratified into high and low SOX17 expression groups (230943_at). *P < 0.05; ****P < 0.0001; ns not statistically significantly different.

A comparative analysis of miR-200a expression in normal versus different types of cancerous tissues with data available in The Cancer Genome Atlas (TCGA) database showed that among 33 different tumors, miR-200a expression was upregulated in 14 different cancers including lung cancer (Supplementary Fig. 7A). miR-200a was one of the top 20 miRs, overexpressed in LUAD (Supplementary Fig. 7B). Functional Enrichment Analysis of miR-200a targets by miRTarBase 2020 revealed that LUAD falls among 30 enriched pathways modulated by miR-200a in different cancers (Supplementary Fig. 7C). miR-200a expression was significantly up-regulated in LUAD tissues as compared to adjacent normal in TCGA samples (Fig. 7A). We also found a significant increase of miR-200a level in individual LUAD stages with respect to normal (Fig. 7B). Moreover, in every nodal metastatic status miR-200a expression was significantly up-regulated (Fig. 7C). Additionally, miR-200a expression was analyzed in the sera of healthy versus lung cancer patients using the Selected Circulating miRNome Dataset which indicated a significant rise in miR-200a level in lung cancer patients’ sera (Fig. 7D). miR-200a expression in the sera of lung cancer patients was also shown to be higher as compared to the sera of non-cancerous other lung diseases (Fig. 7E).

Comparative analysis of SOX17 gene expression in normal and different cancerous tissues was performed with RNA sequencing data available in TCGA database. Differential expression analysis demonstrated that SOX17 expression was downregulated in 15 different cancerous tissues including LUAD as compared to normal (Supplementary Fig. 8A). Analysis of SOX17 gene expression by TCGA database further revealed that in primary LUAD tumors SOX17 expression was significantly decreased with respect to normal (Fig. 7F). Next, we analyzed the expression of SOX17 gene in different stages of LUAD tissues versus normal and observed that in all LUAD stages SOX17 were significantly reduced (Fig. 7G). In different LUAD nodal metastasis levels, a significant downregulation of SOX17 expression was observed (Fig. 7H). Next, we performed another comparative analysis of SOX17 protein expressions in different cancer versus adjacent normal tissues with the data available in The Clinical Proteomic Tumor Analysis Consortium (CPTAC) database. The data suggested that SOX17 expression was significantly downregulated in only lung cancer (Supplementary Fig. 8B). Further analysis also revealed that in LUAD samples SOX17 protein level was significantly downregulated than normal (Fig. 7I). Moreover, excepting grade I LUAD, in every other grade SOX17 expression was significantly lower as compared to normal (Fig. 7J). Consistent with these findings, we also observed by Kaplan-Meier analysis that low SOX17 expression was associated with adverse prognosis and poor patients’ survival (Fig. 7K).

### aPC-EVs via miR-200a transfer promote proliferation, metastasis, and chemotherapeutic resistance in vivo

**Fig. 8:**
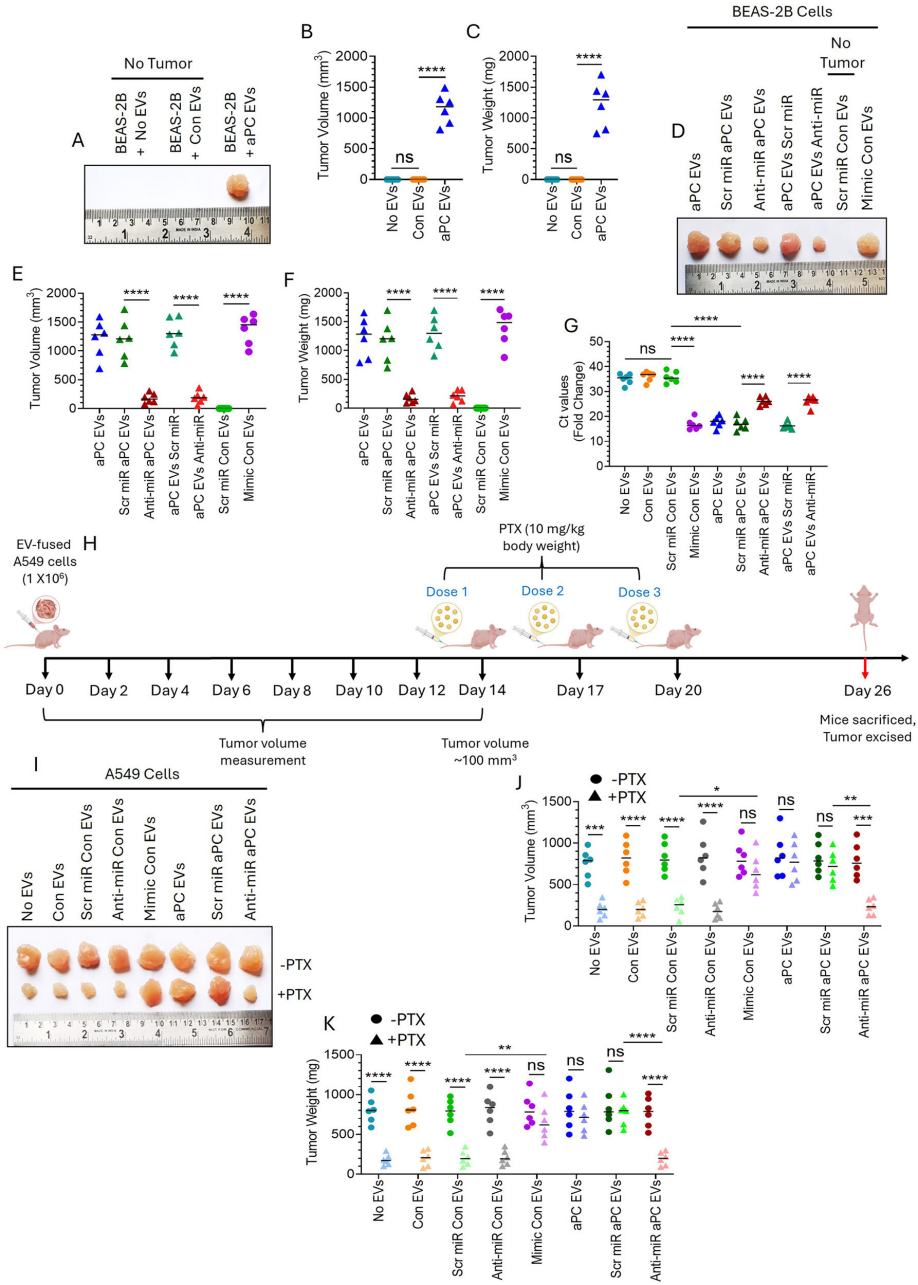
aPC-released EVs promote proliferation, metastasis, and confer chemoresistance in vivo via miR-200a transfer. **A** A549 cells were treated with a CV or aPC for 16 h. EVs were isolated from the supernatant and incubated with BEAS-2B cells (cells:EVs = 1:100) in equal number (1 × 10^8^) for 4 h. The EV-fused BEAS-2B cells (1 × 10^6^) were resuspended in 200 µL of serum-free media with Matrigel at 1:1 ratio and injected into right flanks of BALB/c nude mice via subcutaneous injection. After 25 days, mice were euthanized, tumors were excised and imaged. **B** Tumor volume (after measuring primary tumor in three dimensions, a, b, c and volume were calculated using the formula: *abc* X 0.52) and **C** tumor weight was measured. **D** A549 cells were transfected with Scr miR or Anti-miR followed by the treatment with aPC. The EVs so generated were fused with BEAS-2B cells in equal number (1 × 10^8^; aPC EVs, Scr miR aPC EVs and Anti-miR aPC EVs group). Alternatively, BEAS-2B cells were pre-transfected with Scr miR or Anti-miR followed by the fusion of aPC-EVs (aPC EVs Scr miR and aPC EVs Anti-miR group). Again, EVs isolated from Scr miR or miR Mimic-transfected A549 cells were fused with BEAS-2B cells in equal number (1 × 10^8^); Scr miR Con EVs and Mimic Con EVs group). The EV-fused BEAS-2B cells were introduced into the mice as mentioned in (**A**). After the indicated time, mice were sacrificed to isolate the tumor and imaged. Accordingly, **E** tumor volume and **F** tumor weight was measured. **G** EVs generated from A549 cells after various treatments as mentioned in (**D**) were fused with BEAS-2B cells in equal number (1 × 10^8^). The EV-fused cells were administered into mice via the tail vein. After 25 days, mice were euthanized, lungs were harvested, and metastatic burden of BEAS-2B cells was analyzed as mentioned briefly in Materials and Methods. **H** EVs released from A549 cells after different treatments as described in (**D**) were fused with naïve A549 cells in equal number. The EV-fused A549 cells (1 × 10^6^) were injected subcutaneously into the right flanking region of the mice. Tumor size was monitored every alternative day. After 14 days, when the tumor size became ~100 mm^3^ the mice were given PTX (10 mg/kg body weight) through tail vein every three days (three times). After 12 days, mice were sacrificed, tumor was isolated, and **I** image was taken. **J** Tumor volume and **K** tumor weight was calculated. *P < 0.05; **P < 0.01; ***P < 0.001; ****P < 0.0001; ns not statistically significantly different.

An equal number of EVs (CV- or aPC treated) from A549 cells was fused with BEAS-2B cells. The EV-fused cells were injected into the BALB/c nude mice via subcutaneous injection. After 25 days, the mice were euthanized, and tumors were exercised to measure tumor volume and weight. No visible tumor was obtained in control- or CV-EV-fused BEAS-2B cells injected groups (Fig. 8A–C). In contrast, aPC-EV-fused BEAS-2B showed visible tumors (Fig. 8A–C). Next, to determine the role of miR-200a in aPC-EV-induced proliferation of BEAS-2B in vivo, A549 cells were transfected with a scrambled miR or anti-miR-200a followed by aPC treatment. An equal number of EVs were fused with BEAS-2B cells and analyzed for tumor volume and weight. A549 cells were also transfected with a scrambled miR or miR-200a mimic followed by EV isolation and analyzing for BEAS-2B tumors after EV fusion. Alternatively, BEAS-2B cells were transfected with a scrambled miR or anti-miR-200a followed by fusion of aPC EVs and tumor volume and weight monitored. The data showed that aPC-EV-induced BEAS-2B tumor volume and weight were significantly downregulated upon introduction of anti-miR 200a, as expected, scrambled miR did not alter tumor size (Fig. 8D–F). However, miR-200a mimic incorporated control EVs showed similar tumor size as that of aPC-EVs (Fig. 8D–F). To determine the metastatic potential, EV-fused BEAS-2B cells were administered into the mice via the tail vein. After 25 days, mice were euthanized, lungs were harvested and analyzed for BEAS-2B lung colonization (Fig. 8G). The data revealed that as compared to control EVs, aPC-EV-fused BEAS-2B cells showed more metastatic potential which was completely abrogated upon the introduction of anti-miR 200a (Fig. 8G). miR-200a mimic packaged control EVs also showed similar metastatic potential to that of aPC-EVs (Fig. 8G). To test the drug resistance properties, EVs released from A549 cells were fused with naïve A549 cells in equal numbers. EV-fused A549 cells were introduced into the mice via subcutaneous injection. After 14 days, when the tumor volume reached ~100 mm^3^, mice were administered with PTX (10 mg/kg body weight) through tail vein every three days (three times). After 12 days, mice were sacrificed, tumors were excised, tumor volume and weight were measured (Fig. 8H). Treatment of PTX significantly reduced tumor volume and weight in every treatment group except for mimic con EVs, aPC EVs, and Scr miR aPC EVs which showed resistance to PTX (Fig. 8I–K). However, the incorporation of anti-miR 200a reversed the resistance effect of aPC-EVs signifying that aPC-EVs via miR-200a transfer confer A549 cells resistance against PTX (Fig. 8I–K).

## Discussion

An enhanced expression of EPCR [18] and PAR1 [19] is observed in LUAD, shown to be associated with disease pathogenesis and poor survival [18, 19]. Although aPC signaling plays a key role in human LUAD cells [18], the underlying mechanisms are not fully elucidated. In the present study, for the first time, we provide mechanistic insight into aPC-driven human LUAD progression. Here, we show that exposure of human LUAD lines with aPC results in a significant increase in EV generation depending on EPCR-PAR1 signaling. Mechanistically, aPC-EPCR-PAR1 signaling induces the sequential activation of intracellular RhoA, ROCKII, JNK1/2, and MLC2 to alter myosin dynamics, thereby facilitating EV release. Equal number of aPC-EVs, but not control EVs, via miR-200a transfer promote proliferation, migration, and invasion of normal lung epithelial cells while conferring lung cancer cells resistance against chemotherapeutic drug, PTX. The analysis of miR-200a expression in equal number of control- versus aPC-EVs delineates that aPC-EVs retain significant amount of miR-200a as compared to control EVs. This is due to the induced expression of miR-200a in the cells by aPC stimulation and consequently increased packaging of miR-200a into the EVs. The incorporation of aPC-EVs, unlike the control EVs, enhances the level of miR-200a in the recipient cells, thereby triggering the phenotypic alteration. This enhancement of miR-200a expression is due to the transfer of miR-200a through the EVs but not the EV-induced endogenous expression of miR-200a. It is noteworthy that although we did not find any effect of the control EVs in our experimental concentration, at a relatively higher concentration the control EVs also show similar phenotypic responses to that of aPC-released EVs, probably via the transfer of significant amount of miR-200a into the recipient cells (data not shown). aPC-EV-mediated delivery of miR-200a targets SOX17 in recipient cells, thereby promoting lung tumorigenesis. SOX17 downregulation is also observed in human lung cancer tissues associated with poor survival. Our in vivo studies further demonstrate that aPC-EV-fused normal lung epithelial cells, via miR-200a transfer, promote tumor development and metastasis whereas aPC-EV-fused lung cancer cells show resistance against PTX.

Earlier studies have indicated that the RhoA-ROCKII axis plays an important role in the metastasis of human lung cancer [40, 41]. As aPC is also known to promote lung cancer metastasis [18], we analyzed the possibility of RhoA-ROCKII axis in promoting aPC-induced release of EVs from human lung cancer cells. As predicted, we observed a significant inhibition of aPC-mediated EV generation upon perturbation of either RhoA or ROCKII. ROCKII triggers JNK1/2 phosphorylation [33] which often promotes lung cancer migration and invasion via enhancing MMP-2 and -9 [42]. The inhibition of JNK1/2 also abrogated aPC release of EVs from lung cancer cells. JNK1/2 activation further promotes MLC2 phosphorylation [32] which essentially regulates myosin activity to induce EV release [30, 31]. Consistent with these, we also observed a significant decrease in aPC-induced EV release upon perturbation of MLC2. However, upon determining the chronology of activation we conclude that aPC signaling promotes RhoA activation which further activates ROCKII. ROCKII, through JNK1/2 phosphorylation (activation) ultimately leads to the activation of MLC2 via phosphorylation. Phospho-MLC2 plays a crucial role in altering myosin dynamics, facilitating EV release (Supplementary Fig. 9).

EVs carry bioactive molecules such as proteins, lipids, metabolites, mRNAs, miRs etc. and upon delivering them alter phenotypes of the recipient cells [43–45]. Among all the EV-transported biomolecules, miRs appear to be most important in altering the expression of proteins in recipient cells [46, 47]. Emerging evidence suggests that miR-200a plays a key role in the development and progression of lung cancer. Studies from Guo et al. have indicated that miR-200a expression is significantly elevated in the early stages of LUAD as compared to normal lung tissues which favor tumor spheroid growth of LUAD [48]. Mechanistically, the group has delineated that miR-200a inactivates S6K which leads to the induction of S6K substrate, IRS-1 [48]. Enhanced IRS-1 expression is essential in the activation of AKT which further promotes tumor spheroid growth [48]. In another study, miR-200a is shown to induce proliferation and metastasis of non-small cell lung cancer (NSCLC) cells by targeting SOX17 [37]. SOX17 over-expression reverses miR-200a-induced NSCLC proliferation and metastasis [37], identifying miR-200a as a potential candidate for targeting NSCLC progression. Moreover, a long non-coding RNA, FTX is shown to sponge miR-200a thereby impairing the inactivation of miR-200a target, FOXA2, a transcription factor, acting as a tumor suppressor that attenuates tumor cell proliferation, migration, and invasion [49]. In NSCLC, FTX expression is down-regulated, which leads to miR-200a-mediated inactivation of FOXA2, thereby promoting NSCLC proliferation and metastasis [49]. A higher miR-200a expression in the peripheral blood of NSCLC patients also exhibits high clinical diagnostic efficacy with increased risk of death of NSCLC patients [50]. In a recent study, the expression of miR-200a is shown to be elevated in the plasma EVs of pancreatic ductal adenocarcinoma (PDAC) as compared to the benign form which appears to be a clinically useful diagnostic biomarker for PDAC [51]. However, whether EV-encapsulated miR-200a plays any role in lung cancer pathogenesis, remains ill-defined. In the present study, we observed a significant up-regulation of miR-200a expression in the tissues and sera of lung cancer patients as compared to normal in the TCGA samples. The above findings raise the possibility that miR-200a may play a role in pathogenesis of lung cancer.

SOX17 contains an evolutionary conserved 3’-UTR sequence which appears to be the binding region for miR-200a [37–39]. Consistent with these findings, we also observed that fusion of miR-200a-loaded EVs to recipient cells results in the downregulation of SOX17 and inhibition of miR-200a restores SOX17 expression. Furthermore, direct knockdown of SOX17 in recipient cells also promotes cell proliferation, migration, invasion, and apoptosis resistance without the involvement of EVs. Like our findings, we also observed a significant downregulation of SOX17 expressions both in mRNA and protein level in human LUAD tissues as compared to adjacent normal in TCGA and CPTAC samples respectively, and low SOX17 expression is associated with poor survival of LUAD patients.

Consistent with these results, our in vivo data shows that incorporation of aPC-EVs from lung cancer cells into normal lung epithelial cells resulted in the formation of visible tumors in BALB/c nude mice. Administration of normal lung epithelial cells alone did not form any such tumors, nor control EV-fused cells. Moreover, the introduction of anti-miR 200a dramatically reduced the size of aPC-EV-fused BEAS-2B tumors. On the other hand, miR-200a mimic-packaged control EVs from lung cancer cells again showed tumors of normal lung epithelial cells. These results were consistently observed in our metastatic studies in vivo. aPC-EV-fused normal lung cancer cells develop significant metastatic burden which was downregulated upon introduction of anti-miR 200a. Again, miR-200a mimic-incorporated control EVs developed a similar metastatic burden to that of aPC-EVs. These indicate that aPC-EVs from lung cancer cells transform normal lung epithelial cells into pro-cancerous, augmenting in the spread of disease. Additionally, aPC-EVs from lung cancer cells conferred lung cancer resistance to chemotherapeutic drug, PTX in vivo. Like the above findings, drug resistance was also found to be dependent on miR-200a. These imply that aPC-EVs from human lung cancer cells promote proliferation, migration, invasion, and drug resistance to recipient cells via miR-200a transfer. Interestingly, the incorporation of miR-200a-laden EVs into naïve lung cancer cells did not show any increase in cell proliferation. The possible reason behind this could be that the lung cancer cells used in this study, A549, are themselves highly proliferative. Therefore, the effect of miR-200a-loaded EVs, being so small, might not show up. A higher EV concentration may develop visible proliferation in A549 cells.

In summary, our findings provide a novel mechanistic insight into the role of aPC in human lung cancer progression, via the induction of EVs. aPC primarily resides in the blood [52], however, upon binding to endothelial EPCR aPC gets transcytosed from the lumen of blood vessels to the subendothelial space [22], thereby reaching the tissues and encountering tumor cells [23]. Our current study shows that aPC binds to EPCR on lung cancer cells, thereby activating PAR1 and aPC-EPCR-PAR1 signaling results in EV release from lung cancer cells. We also observed that the RhoA-ROCKII-JNK1/2-MLC2 pathway plays a major role in aPC-induced biogenesis of EVs in lung cancer cells. Furthermore, our study delineates that aPC-EVs are enriched with miR-200a, which upon fusion of EVs with the normal lung epithelial cells reaches to the cytosol of recipient cells, thereby targeting SOX17, leading to the induction of cell proliferation, migration, and invasion. aPC-EV-mediated transfer of miR-200a also imparts lung cancer resistance against chemotherapeutic regimens. Our data suggest that targeting aPC holds promises in the treatment of lung cancer. However, there is a high risk of VTE in lung cancer [3] and being a natural anti-coagulant, targeting aPC directly may lead to severe thrombotic outcomes in lung cancer patients, contributing further to the decreased survival. Therefore, targeting the aPC-induced signaling without compromising its anti-coagulant property becomes indispensable. Hence, targeting the biogenesis of EVs, EVs’ driving factors, and EV uptake by the recipient cells open developing novel therapeutic strategies in limiting EV-associated lung cancer progression and thus increasing patients’ survival.

## Supplementary information

Supplementary Information

Original Raw Data_Western blots and qRT-PCR

## Data Availability

All the sequencing data generated is accessible at the Gene Expression Omnibus (GEO) in NCBI via GEO Series accession number GSE303686.
