# Supplementary material for: Activated protein C promotes human lung cancer progression through the release of tumor extracellular vesicles and transfer of microRNA-200a
Source: Cell Death Dis. 2025 Nov 21;16(1):848. doi: 10.1038/s41419-025-08173-z (PMC12638984; doi:10.1038/s41419-025-08173-z)
Supplement: Supplementary file 1 — Supplementary Information [file 41419_2025_8173_MOESM1_ESM.docx]

**Activated protein C promotes human lung cancer progression through the release of tumor extracellular vesicles and transfer of microRNA-200a**

Madhura Chatterjee,^1,2^ Deepak Parashar,^3^ Rajan Pandey,^4,5^ Tanmoy Mukherjee,^6^ Saurabh Gupta,^7^ Subhojit Paul,^8^ Akash Chatterjee,^8^ Gunjan Potale,^1^ Prity Dhara,^1^ V. V. Sathibabu Uddandrao,^9,10^ S. Sengottuvelu,^11^ Aishwarya Sharma,^12^ Umesh Kumar,^13^ Jhansi Magisetty,^14^ Arindam Maitra,^1,^* Kaushik Das^1,^*

^1^Biotechnology Research and Innovation Council-National Institute of Biomedical Genomics, Kalyani, West Bengal, India

^2^Regional Centre for Biotechnology, Ph.D. Program, India

^3^Division of Hematology & Oncology, Department of Medicine, Medical College of Wisconsin, Milwaukee, WI, USA

^4^Translational Bioinformatics Group, International Centre for Genetic Engineering and Biotechnology, New Delhi, India

^5^Department of Physiology, University of Arizona, Tucson, AZ, USA

^6^Clapp & Mayne Global Health Sector Consulting Group and Renaissance Information Systems (CAMRIS) International (under Contract No. 75N93019D00025 with National Institute of Allergy and Infectious Diseases), NIH, DHHS, Rockville, MD, USA

^7^Department of Biotechnology, GLA University, Mathura, Uttar Pradesh, India

^8^School of Biological Sciences, Indian Association for the Cultivation of Science, Kolkata, India

^9^Department of Biotechnology, Karpagam Academy of Higher Education (Deemed to be University), Coimbatore, Tamil Nadu, India

^10^Centre for Active Pharmaceutical Ingredients, Karpagam Academy of Higher Education (Deemed to be University), Coimbatore, Tamil Nadu, India

^11^Department of Pharmacology, Nandha College of Pharmacy, Erode, Tamil Nadu, India

^12^Department of General Surgery, Sree Balaji Medical College and Hospital, Chromepet, Chennai, India

^13^University Institute of Engineering, Chandigarh University, NH5, Gharuan, Chandigarh-Ludhiana Highway, Mohali, Punjab, India

^14^Department of Zoology, Central University of Punjab, Bathinda, India

*Correspondence:

[kd3@nibmg.ac.in](mailto:kd3@nibmg.ac.in) (K.D.), ORCID: https://orcid.org/0000-0003-0386-0543

[am1@nibmg.ac.in](mailto:am1@nibmg.ac.in) (A.M.), ORCID: https://orcid.org/0000-0002-0249-8856

Biotechnology Research and Innovation Council-National Institute of Biomedical Genomics, Kalyani 741251, West Bengal, India

**Supplementary Materials and Methods**

**Reagents**

The human aPC used in the current study was purchased from Sigma-Aldrich. The PKH67 dye, Actinomycin D, and Paclitaxel (PTX) were also purchased from Sigma-Aldrich. The exosome panel kit which includes antibodies against human TSG101, Calnexin, HSP70, CD81, CD9, and CD63 as well as Caspase 3 Assay Kit was obtained from Abcam (Cat. No. ab275018). Rabbit polyclonal antibody against human EPCR was purchased from ThermoFisher Scientific (Cat. No. PA5-32217). Mouse monoclonal antibody against human PAR1 (ATAP2; Cat. No. sc-13503) and JNK1/2 (Cat. No. sc-137019) were procured from Santa Cruz Biotechnology. Mouse monoclonal antibody against human GAPDH was obtained from Bio-Rad (MCA4740). Rhosin (RhoA inhibitor), SP600125 (JNK inhibitor/JNK I) and Y27632 (ROCK Inhibitor) were purchased from Sigma-Aldrich. Polyclonal antibody against phospho-JNK1/2 antibody (p-Thr183/p-Tyr185) (Cat. No. J4644) was purchased from Sigma-Aldrich. Rabbit polyclonal antibodies against human phospho-MLC2 (Cat. No. 3671), MLC2 (Cat. No. 3672), and Bax (Cat. No. 2772) as well as monoclonal antibody against human Bcl-2 (Cat. No. 3498) were obtained from Cell Signaling Technology. carboxyfluorescein diacetate succinimidyl ester (CFSE) Cell Proliferation Kit was purchased from Invitrogen. Clarity Western ECL substrate was obtained from Bio-Rad. Crystal violet solution and MTT were procured from HiMedia. Rabbit polyclonal antibody against human SOX17 (Cat. No. ITT5055) was purchased from G-Biosciences. Chromogenic substrate for aPC (S-2366) was obtained from DiaPharma. RhoA activation assay kit was purchased from Abcam (Cat. No. ab211164). Flow Cytometry Sub-Micron Particle Size Reference Kit was obtained from Invitrogen (F13839).

**Cells**

The human lung epithelial adenocarcinoma cell lines, A549 and HCC827 were kindly gifted by Prof. Prosenjit Sen, Indian Association for the Cultivation of Science, Kolkata, West Bengal, India and maintained in F-12K and RPMI-1640 media, respectively supplemented with 10% fetal bovine serum (FBS; Gibco) and 1X penicillin-streptomycin solution (Invitrogen). The normal lung epithelial cell line, BEAS-2B was purchased from ATCC (Cat. No. CRL 3588) and cultured in airway epithelial cell basal medium (ATCC; Cat. No. PCS-300-030) with epithelial cell growth supplement (ATCC; Cat. No. PCS-300-040). The cells were grown at 37°C with 5% CO_2_ in a humidified atmosphere.

**Primer, miR inhibitor, and miR mimic sequences**

Human miR-181a-2: reverse transcriptase (RT) primer: 5’-GAAAGAAGGCGAGGAGCAGATCGAGGAAGA AGACGGAAGAATGTGCGTCTCGCCTTCTTTCGGTACAGT-3’, forward primer: 5’-ACCACTGACCGTTGACTG-3’, reverse primer: 5′-CGAGGAAGAAGACGGAAGAAT-3′; human miR-200a: RT primer: 5’-GAAAGAAGGCGAGGAGCAGATCGAGGAAGA AGACGGAAGAATGTGCGTCTCGCCTTCTTTCACATCGTT-3’, forward primer: 5’-GCTAACACTGTCTGGTAACG-3’, reverse primer: 5′-CGAGGAAGAAGACGGAAGAAT-3′; human miR-200b: RT primer: 5’-GAAAGAAGGCGAGGAGCAGATCGAGGAAGAAGACGGAAGAATGTGCGTCTCGCCTTCTTTCTCATCATT-3’, forward primer: 5’-GCGTAATACTGCCTGGTAATG-3’, reverse primer: 5′-CGAGGAAGAAGACGGAAGAAT-3′; human miR-365b: RT primer: 5’-GAAAGAAGGCGAGGAGCAGATCGAGGAAGA AGACGGAAGAATGTGCGTCTCGCCTTCTTTCATAAGGAT-3’, forward primer: 5’-GCGTAATGCCCCTAAAAATCC-3’, reverse primer: 5′-CGAGGAAGAAGACGGAAGAAT-3′; human miR-212: RT primer: 5’-GAAAGAAGGCGAGGAGCAGATCGAGGAAGAAGACGGAAGAATGTGCGTCTCGCCTTCTTTCAGTAAGCA-3’, forward primer: 5’-ACCTTGGCTCTAGACTGC-3’, reverse primer: 5′-CGAGGAAGAAGACGGAAGAAT-3′; human miR-615: RT primer: 5’-GAAAGAAGGCGAGGAGCAGATCGAGGAAGA AGACGGAAGAATGTGCGTCTCGCCTTCTTTCAAGAGGGA-3’, forward primer: 5’- TCCGAGCCTGGGTC-3’, reverse primer: 5′-CGAGGAAGAAGACGGAAGAAT-3′; human miR-4521: RT primer: 5’-GAAAGAAGGCGAGGAGCAGATCGAGGAAGAAGACGGAAGAATGTGCGTCTCGCCTTCTTTCCTGAGCAC-3’, forward primer: 5’-GCTAAGGAAGTCCTGTGC-3’, reverse primer: 5′-CGAGGAAGAAGACGGAAGAAT-3′, human U6: RT primer: 5’-CGCTTCACGAATTTGCGTGTCA-3’, forward primer: 5’-GCTTCGGCAGCACATATACTAAAAT-3’, reverse primer- 5’-CGCTTCACGAATTTGCGTGTCAT-3’; human c-myc: forward primer: 5’-ATGAAAAGGCCCCCAAGGTA-3’, reverse primer: 5’-CGTTTCCGCAACAAGTCCTCT-3’; human HK2: forward primer: 5’-CCAGTTCATTCACATCATCAG-3’, reverse primer: 5’-CTTACACGAGGTCACATAGC-3’; human 18S rRNA: forward primer: 5’-CGGCGACGACCCATTCGAAC-3’, reverse primer: 5’-GAATCGAACCCTGATTCCCCGTC-3’; miR-200a mimic and inhibitor (anti-miR): Scrambled miR-200a: 5’-UUCUCCGAACGUGUCACUGUU-3’, miR-200a mimic: 5’-UAACACUGUCUGGUAACGAUGU-3’, anti-miR 200a: 5’-ACAUCGUUACCAGACAGUGUUA-3’; anti-miR-200b: 5’-UCAUCAUUACCAGGCAGUAUUA-3’; anti-miR-212: 5’-AGUAAGCAGUCUAGAGCCAAGGU-3’; anti-miR-181a-2: 5’-GGUACAGUCAACGGUCAGUGGU-3’; anti-miR-365b: 5’-AUAAGGAUUUUUAGGGGCAUUA-3’.

**Silencing of EPCR, PAR1, or SOX17**

Cells were transfected with siRNA specific for EPCR (5’- GUG GAC GGC GAU GUU AAU UAC TT-3’; 200 nM; Sigma), PAR1 (5’-AGA UUA GUC UCC AUC AAU-3’ and 5’-AGG CUA CUA UGC CUA CUA C-3’; 200 nM; Sigma), or SOX17 (a combination of three double stranded siRNA sequences were used; Seq. 1: sense: 5’-GAGCUAAGCAAGAUGCUAGtt-3’, antisense: 5’-CUAGCAUCUUGCUUAGCUCtg-3’; Seq. 2: sense: 5’-GGCUGUUCAAAAAUUUCGGtt-3’, antisense: 5’-CCGAAAUUUUUGAACAGCCtC-3’; Seq. 3: sense: 5’-GAACCCAGAUCUGCACAACtt-3’, antisense: 5’-GUUGUGCAGAUCUGGGUUCtg-3’; 200 nM; Applied Biosystems). Non-specific siRNA (5′-GAU UAU GUC CGG UUA UGU AUU -3′; 200 nM; Sigma) was used as scrambled control. Transfection was performed by using Lipofectamine 3000 Transfection Reagent (Invitrogen) in a serum-free medium. Eight hours following transfection, the serum-free medium was replaced with complete medium, and cells were cultured for 48 h to proceed further for experiments. Knockdown of target genes was determined by immunoblotting.

**Isolation of EVs**

EVs were isolated from culture supernatants as previously mentioned with a few modifications [1-5]. Briefly, the cell culture supernatant was centrifuged at 2,500 X g for 10 mins to pellet down cell debris and apoptotic bodies. After discarding the pellet, the supernatant was again centrifuged at 21,000 X g for 1 h to precipitate the EVs. The EVs were suspended in the original volume of 1X HBSS (Gibco) and re-centrifuged at 21,000 X g for 1 h (washing). After washing twice, EVs were finally suspended in one-fifth of the original volume of HBSS and proceeded further for analysis. All the procedures mentioned above were accurately performed at 4°C. For exosome (Exo) isolation, the EV fraction was separated, and the supernatant was further centrifuged at 1,00,000 X g for 1 h to pellet down the Exos.

**EV quantification by NTA Nano-Sight**

EVs from unlabelled cells were re-suspended in 1X HBSS earlier passed through 0.1 µm of syringe-driven filters (HiMedia) and analyzed by nanoparticle tracking analysis (NTA) using Nano-Sight NS300 (Malvern panalytical, Westborough, MA). EV number and size were determined under the following conditions: camera level, 14 (NTA 3.0 levels), detection threshold, 5, and temperature, 21-23°C.

**Transfection of cells with a plasmid reporter, pEGFP-N1, cloned with CD9**

The PCR amplification of CD9-cDNA was cloned in pEGFP-N1 vector (Clontech Laboratories) to obtain the CD9-EGFP fusion protein construct. A549 cells in the serum free media were transfected with 5 µg of the fusion protein construct by using Lipofectamine 3000 as per the manufacturer’s guideline. Eight hours following transfection, the serum free media was replaced with complete media and the cells were further incubated for another 48 h to proceed for the experiments. After the indicated time, the expression of CD9-EGFP in the cells was analyzed by western blotting with CD9-specific antibody.

**Quantification of the EVs by flow cytometry**

The CD9-EGFP-overexpressed A549 cells were serum starved for 1 h followed by the treatment of a CV or aPC for 16 h. After the indicated time, the EVs in the supernatant were isolated by differential centrifugation and quantified by flow cytometry (BD FACSAria III). Briefly, the EV samples were acquired with a reduced threshold of 2000. Area for acquisition was standardized earlier by using fluorescent beads of different sizes on the 488 nm laser channel. EVs (CD9-GFP^+^) were analyzed by the emission on the 488 nm laser channel after normalizing for the volume. Gating strategy was performed by using FlowJo (BD biosciences).

**Imaging of the EVs by TEM**

EVs isolated as described above were suspended in 100 µL of 1X phosphate buffered saline (PBS; 0.137 M NaCl, 0.0027 M KCl, 0.01 M Na_2_HPO_4_, 0.0018 M KH_2_PO_4_; pH 7.4) and placed on a copper grid which was previously coated with Formvar in chloroform (0.125%). The grids were then stained with 1% (v/v) uranyl acetate in deionized water and imaged under ultra-high resolution field emission gun transmission electron microscope (UHR FEG TEM; IACS, Kolkata).

**EV quantification by western blotting**

Both the isolated EVs and EV-producing cells were lysed in 1X sodium dodecyl sulfate-polyacrylamide gel electrophoresis (SDS-PAGE) sample buffer followed by immunoblotting and probed for TSG101, Calnexin, HSP70, CD81, CD9, and CD63. Band intensities were quantified by densitometric analysis and graphical representation was made to quantify the EVs after normalization with respective cell lysates.

**Western blot analysis**

Western blot analysis was performed as previously mentioned [6] with a few minor modifications. Briefly, cells were lysed with 1X SDS-PAGE sample buffer and heated at 95°C for 5 min. Protein separation was carried out by SDS-PAGE and the separated proteins were transferred onto polyvinylidene difluoride membrane (PVDF). The membrane was blocked with 5% non-fat, dried milk in 1X Tris Buffer Saline (TBS; Tris: 20 mM, NaCl: 137 mM) for 1 h and probed with primary antibody (with the recommended dilutions) overnight at 4°C. On the following day, the membrane was washed with TBST (1X TBS with 0.1% Tween 20; 4 washes, 5 min each wash) and incubated with secondary antibody (dilution 1:10,000; horseradish peroxidase-conjugated; Bio-Rad) for 1 h. After TBST washing (4 washes, 20 min each wash), the membrane was developed using the enhanced chemiluminescence (ECL) method. Band intensity was measured by densitometric analysis.

**RhoA activation assay**

The activation of RhoA was determined by using the RhoA activation assay kit as per the manufacturer’s guidelines. In brief, Rhotekin RBD (Rho binding domain) agarose beads were selectively used to isolate and pull-down the active form (GTP bound state) of Rho from the cell lysates. The precipitated GTP-RhoA is detected by western blotting by using a human anti-RhoA specific monoclonal antibody.

**Enrichr-Jensen DISEASES analysis**

Potential gene targets of selected differentially expressed miRNAs were analysed using miRDB, a microRNA target prediction database. Separate lists of genes identified through miRDB were further annotated for their association with diseases using Enrichr [7-9]. Jensen DISEASES, which is an Enrichr feature that emphasizes links between diseases and genes through text mining, was used to enrich for disease terms and expressed in terms of abundance and p-value < 0.05.

**Uptake of EVs by the recipient cells**

A549 cells, grown to confluency on 6-well culture dishes (two wells together for each treatment), were exposed to PKH67 dye (20 mM; Sigma) for 30 mins at 37°C followed by washing twice with 1X HBSS. After labelling with PKH67, cells in the 6-well culture dishes were treated with a control vehicle (CV) or aPC (25 nM) for 16 h. The EVs were isolated from the culture supernatant and incubated in equal number with the recipient cells, BEAS-2B (cells:EVs=1:100) for 4 h at 37°C. After washing twice with 1X HBSS to remove the unbound EVs, the cells were fixed and permeabilized with 4% PFA containing 0.025% Triton X-100. To identify the intracellular localization of endocytosed EVs (green), the permeabilized cells were immunostained with anti-EEA1 antibody (early endosomal antigen 1, an early endosomal marker protein, appeared red in the image). After washing, the nuclei were stained with DAPI for 30 min at room temperature. The appearance of yellow dots (upon co-localization green EVs with red early endosomes) confirms the uptake of EVs by the recipient cells.

**CFSE cell proliferation assay**

BEAS-2B cells were grown on 6-well culture dishes (two wells together for each treatment) and treated with CFSE (10 µM) for 20 min at 37°C. After washing twice with 1X HBSS, cells were incubated with a CV or an equal number of CV-EVs or aPC-EVs from A549 cells (cells:EVs=1:100) for 4 h at 37°C. After washing again with 1X HBSS for two times to remove the unbound EVs, the cells were incubated further for 20 h at 37°C following which proliferation analysis was performed using flow cytometry (BD FACSAria III). When cells labelled with CFSE divide, the fluorescent CFSE dye is distributed among the daughter cells equally. Consequently, the fluorescence intensity/cell decreases with each successive division which was measured by flow cytometry.

**BrdU cell proliferation assay**

BEAS-2B cells grown in 6-well culture dishes (two wells together for each treatment) were fused with equal number (cells:EVs=1:100) of CV-EVs or aPC-EVs from A549 cells followed by culturing in a medium containing 5-bromo-2-deoxyuridine (BrdU; 10 µM; Sigma) for 8 h. Cells were then fixed with 4% paraformaldehyde, washed twice with 1X HBSS, and permeabilized with 0.01% Triton X-100. After blocking with 5% BSA, the cells were incubated overnight with anti-BrdU antibody (Sigma) at 4°C. After washing, cells were probed with an HRP-conjugated secondary antibody for 1 h followed by the addition of tetramethylbenzidine (TMB; Sigma) substrate with H_2_O_2_. O.D. was measured at 450 nm to determine the degree of cell proliferation.

**Transwell migration assay**

EV-fused BEAS-2B cells were seeded in a serum-free media on top of a trans-well membrane (0.8 µm pore size, HiMedia) while the bottom compartment was filled with FBS-containing media. After incubation at 37°C for 24 h, cells on the upper surface of the membrane were scraped off and cells which were migrated through the pores in the lower surface of the membrane were stained with crystal violet (CV) solution (0.1% CV, 0.1 M borate, and 2% ethanol). Images were taken in a bright-field microscope (Radical Scientific Equipments Pvt. Ltd.) of six different fields chosen randomly from which number of migrated cells was quantified.

**Matrigel invasion assay**

Matrigel (Sigma) was coated on top of a trans-well membrane (0.8 µm pore size) and allowed to polymerize at 37°C for 30 min. EV-fused BEAS-2B cells were seeded on top of the Matrigel in a serum-free media while the bottom compartment was filled with FBS-containing complete media. After 48 h, cells on the upper surface of the membrane were removed and the cells invaded through the Matrigel into the lower surface of the membrane were stained with CV solution. Images of six different fields randomly chosen were captured in a bright-field microscope (Radical Scientific Equipments Pvt. Ltd.) from which number of cells invaded was quantified.

**MTT cell viability assay**

A549 cells grown in 96-well culture plates close to confluency were treated with PTX for 48 h. Media was then replaced with 200 μL of fresh media containing 0.5 mg/mL 3-(4,5-dimethylthiazol-2-yl)-2,5-diphenyltetrazolium bromide (MTT) and the cells were incubated at 37°C for 4 h. The supernatant was removed, and the insoluble formazan crystals were solubilized by adding 200 µL of DMSO for 10 min at 37°C. The O.D. was measured at 570 nm from which % cell viability was calculated.

**Caspase 3/7 activity assay**

The EV-fused A549 cells were treated with PTX for 24 h, the cells were lysed with 50 µL of chilled cell lysis buffer (Abcam), and subjected to centrifugation at 10,000 g for 5 min. After discarding the pellet, the supernatant was mixed with 50 μL of 2X reaction buffer (containing 10 mm DTT, Abcam) followed by the addition of Ac-DEVD-pNA substrate (Abcam) at 37°C for 90 min. The amount of pNA released upon cleavage by active caspase3/7 was estimated by measuring the O.D. at 405 nm.

**miR isolation and analysis of expression by qRT-PCR**

miRs were isolated from the EVs as well as cells by using mirPremier microRNA Isolation Kit (Sigma). Reverse transcription of the isolated miRs were performed by using the following cycle: 25°C for 5 min, 42°C for 60 min, and 70°C for 5 min. qRT-PCR was performed at 95°C for 3 min, followed by 40 cycles of 95°C for 5 sec and 62°C for 35 sec.

**miRNA Sequencing and Data Processing**

For miR sequencing of the EVs, A549 cells were treated with a control vehicle (CV) or aPC as mentioned above followed by isolation of the EVs and their quantification by NTA Nano-Sight. An equal number of EVs were subjected to miR isolation using mirPremier microRNA Isolation Kit (Sigma), and RNA integrity was assessed by using NanoDrop. Sequencing was performed on the Illumina NovaSeq 6000 platform, generating 50 bp single end reads.

Raw sequencing reads were subjected to quality control using FastQC to assess read quality, GC content, and adapter contamination [10]. Adapter trimming and quality filtering were performed using Trim Galore! (v0.6.6) (http://www.bioinformatics.babraham.ac.uk/projects/trim_galore/), which integrates Cutadapt for adapter removal. Reads with a Phred score below 20 and lengths shorter than 18 nucleotides after trimming were discarded.

High-quality reads were aligned to the human reference genome (hg38) using STAR aligner (v2.7.9a) with parameters optimized for small RNA mapping as previously mentioned [11]. The alignment was guided by miRNA annotations from miRBase (release 22.1) and Ensembl (release 112) to ensure accurate mapping of mature and precursor miRNAs. Post-alignment, read counts were quantified using HTSeq (v0.13.5) in union mode, using the ensembl GTF annotation file [12].

**Gene expression analysis by RT-PCR**

RNA was extracted by using conventional TRIzol (Invitrogen) method. cDNAs were prepared using oligo(dT) primer (GCC Biotech) with the following reaction conditions: 25°C for 5 min, 46°C for 40 min, and 95°C for 60 sec. RT-PCR was performed using the following condition: 95°C for 20 sec, followed by 40 cycles of 95°C for 3 sec, and 60°C for 30 sec.

***In vivo* experiments**

The number of mice per group was initially calculated by power analysis to detect at least a 25% difference between the control and experimental groups with 80% power to detect the difference with a significance level of 5%. We expected about 20 to 25% standard deviation. Therefore, 8 to 12 mice per experimental group was allocated for the study. However, this number may vary depending upon the experimental conditions and in our study, we found significant differences with 6 to 8 mice between our experimental groups. All the experiments and analyses were performed in a blinded fashion.

In metastatic studies, EV-fused BEAS-2B cells (1 X 10^6^) were introduced into the circulation of BALB/c nude mice via the tail vein injection. After 25 days, mice were euthanized, and lungs were harvested. Genomic DNA was isolated from lung harvests followed by analysis with qPCR as described earlier [13]. In brief, lungs were digested with proteinase K at 55°C and genomic DNA was extracted with phenol-chloroform followed by precipitation with isopropyl alcohol and washing with ethanol. A 200-ng aliquot of genomic DNA was subjected to amplification by qPCR with human-specific HK2 gene- and 18S rRNA primers from which lung metastatic burden was estimated from the Ct values.

To understand the role of the EVs in conferring drug resistance, EVs released from A549 cells after various treatment were fused with naïve A549 cells in equal number (cells:EVs=1:100). The EV-fused A549 cells (1 X 10^6^) were injected subcutaneously into the right flanking region of the BALB/c nude mice. Tumor size was monitored every alternative day by using a vernier calliper. After 14 days, when the tumor size became ~100 mm^3^ the mice were given PTX (10mg/kg body weight) through the tail vein in every 3 days (3 times). After 12 days, mice were sacrificed, tumors were isolated and imaged to calculate tumor volume and weight.

**Statistical analysis**

All the *in vitro* experiments mentioned here were performed three times (biological replicates). For the *in vivo* studies, 6 to 8 mice were used in each experimental group. The sample size (n) for each experimental group/condition both *in vitro* and *in vivo* is shown as individual data point in a dot plot. For the patients’ data, the sample size (n) is mentioned in the individual graph. The data presented here were as mean ± standard error of the mean (SEM). The Student’s t-test was used to analyse the statistically significant differences between the two groups. For more than two groups, One-way ANOVA followed by Tukey’s or Dunnett’s post hoc multiple comparison test, was performed to determine the statistical significance.

**References**

1 Das K, Keshava S, Ansari SA, Kondreddy V, Esmon CT, Griffin JH *et al*. Factor VIIa induces extracellular vesicles from the endothelium: a potential mechanism for its hemostatic effect. *Blood* 2021; 137: 3428-3442.

2 Das K, Keshava S, Pendurthi UR, Rao LVM. Factor VIIa suppresses inflammation and barrier disruption through the release of EEVs and transfer of microRNA 10a. *Blood* 2022; 139: 118-133.

3 Das K, Pendurthi UR, Manco-Johnson M, Martin EJ, Brophy DF, Rao LVM. Factor VIIa treatment increases circulating extracellular vesicles in hemophilia patients: Implications for the therapeutic hemostatic effect of FVIIa. *J Thromb Haemost* 2022; 20: 1928-1933.

4 Das K, Keshava S, Mukherjee T, Wang J, Magisetty J, Kolesnick R *et al*. Factor VIIa releases phosphatidylserine-enriched extracellular vesicles from endothelial cells by activating acid sphingomyelinase. *J Thromb Haemost* 2023; 21: 3414-3431.

5 Das K, Keshava S, Kolesnick R, Pendurthi UR, Rao LVM. MicroRNA-10a enrichment in factor VIIa-released endothelial extracellular vesicles: potential mechanisms. *J Thromb Haemost* 2024; 22: 441-454.

6 Das K, Prasad R, Roy S, Mukherjee A, Sen P. The Protease Activated Receptor2 Promotes Rab5a Mediated Generation of Pro-metastatic Microvesicles. *Sci Rep* 2018; 8: 7357.

7 Chen EY, Tan CM, Kou Y, Duan Q, Wang Z, Meirelles GV *et al*. Enrichr: interactive and collaborative HTML5 gene list enrichment analysis tool. *BMC Bioinformatics* 2013; 14: 128.

8 Kuleshov MV, Jones MR, Rouillard AD, Fernandez NF, Duan Q, Wang Z *et al*. Enrichr: a comprehensive gene set enrichment analysis web server 2016 update. *Nucleic Acids Res* 2016; 44: W90-97.

9 Xie Z, Bailey A, Kuleshov MV, Clarke DJB, Evangelista JE, Jenkins SL *et al*. Gene Set Knowledge Discovery with Enrichr. *Curr Protoc* 2021; 1: e90.

10 Andrews, S. (2010) FastQC: A Quality Control Tool for High Throughput Sequence Data.

11 Dobin A, Davis CA, Schlesinger F, Drenkow J, Zaleski C, Jha S *et al*. STAR: ultrafast universal RNA-seq aligner. Bioinformatics. 2013 Jan 1;29(1):15-21.

12 Anders S, Pyl PT, Huber W. HTSeq--a Python framework to work with high-throughput sequencing data. Bioinformatics. 2015 Jan 15;31(2):166-9.

13 Das K, Paul S, Singh A, Ghosh A, Roy A, Ansari SA *et al*. Triple-negative breast cancer-derived microvesicles transfer microRNA221 to the recipient cells and thereby promote epithelial-to-mesenchymal transition. *J Biol Chem* 2019; 294: 13681-13696.

**Supplementary Figures**

**Supplementary Fig. 1. Diameter range of control (CV) and aPC-released EVs as determined by NTA Nano-Sight. (A)** A549 cells grown onto 6-well culture dishes (two wells together for each treatment) were serum starved for 1 h followed by exposure to a control vehicle (CV) or aPC for 16 h. The EVs were isolated from the culture supernatant by differential centrifugation and suspended in 1X HBSS followed by NTA Nano-Sight analysis. A spectrum showing the diameter range of the EVs as measured by NTA Nano-Sight. **(B)** A violin plot showing the mean diameter of the EVs as measured by NTA Nano-Sight. ns, not statistically significantly different.

**
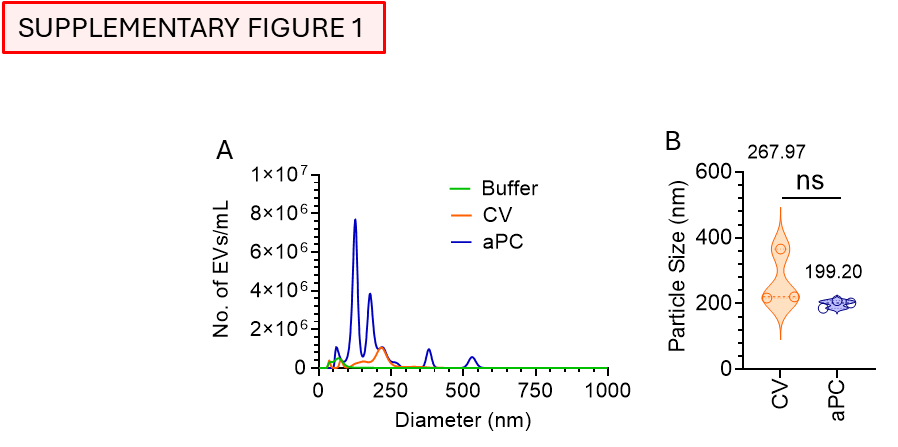
**

**
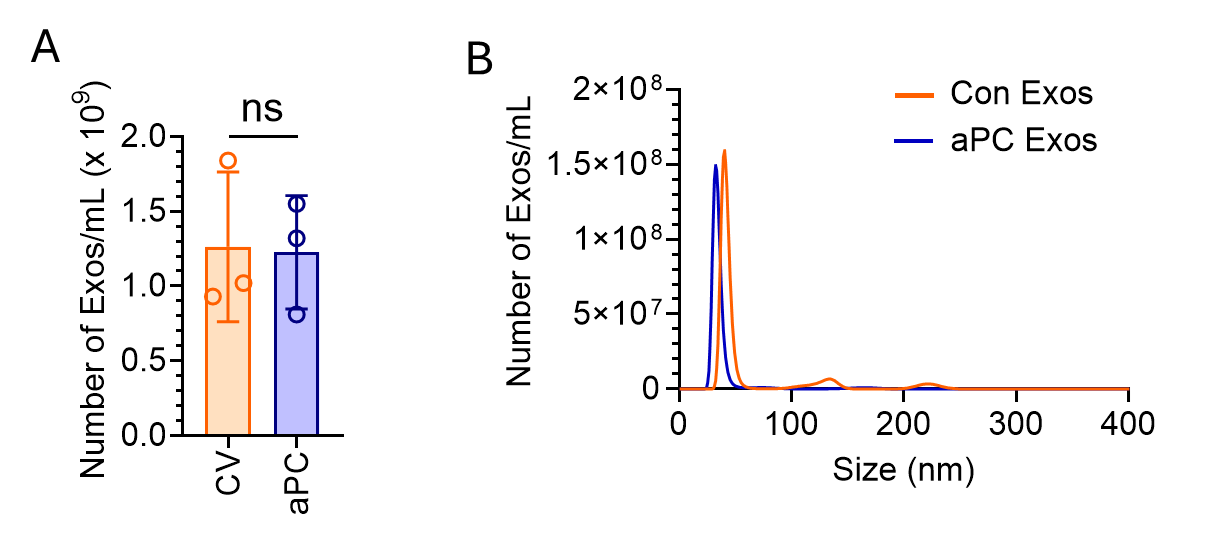
Supplementary Fig. 2. aPC treatment does not induce exosome (Exo) release from lung cancer cells.** A549 cells were treated with a control vehicle (CV) or aPC (25 nM) for 16 h. The culture supernatant was centrifuged at 2,500 X g for 10 mins to precipitate cell debris and apoptotic bodies. After discarding the pellet, the supernatant was again centrifuged at 21,000 X g to pellet down the EVs. After separating the EV pellet, the supernatant was finally centrifuged at 1,00,000 X g to sediment the Exos. After washing twice with 1X HBSS, the Exos were subjected to **(A)** quantification by NTA Nano-Sight. **(B)** Size distribution of the Exos as determined by NTA Nano-Sight. ns, not statistically significantly different.

**Supplementary Fig. 3. aPC triggers EV generation from HCC827 cells.** HCC827 cells were serum starved for 1 h followed by treatment with a CV or aPC (25 nM) for 16 h. EVs were isolated from the culture supernatant and quantified by NTA Nano-Sight. ****P < .0001.


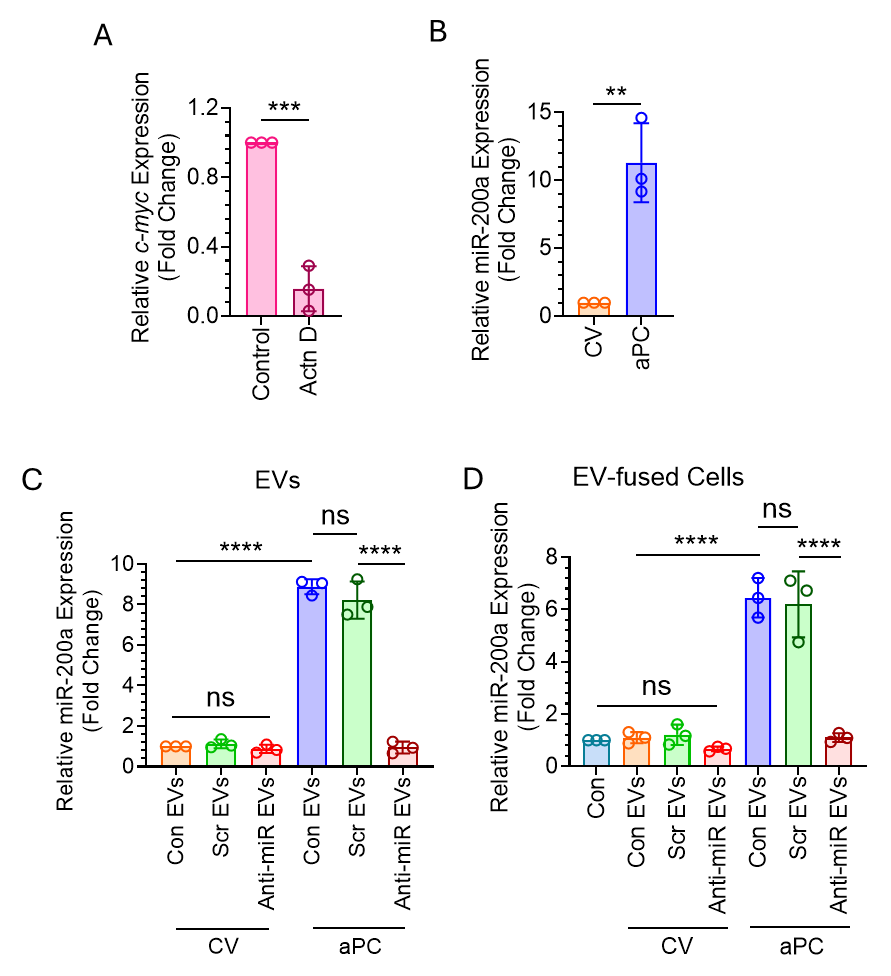
**Supplementary Fig. 4. Treatment of aPC significantly increased the expression of miR-200a in A549 cells and introduction of Anti-miR not only downregulated miR-200a expression in the EVs but also decreased miR-200a level in EV-fused recipient cells. (A)** Actinomycin D effectively reduced the transient expression of *c-myc* in BEAS-2B cells. BEAS-2B cells were challenged with a CV or Actinomycin D (Actn D; 10 µg/mL) for 8 h. The cells were lysed and the expression of *c-myc* was analyzed by real-time PCR. **(B)** A549 cells were treated with a CV or aPC (25 nM) for 16 h. The expression of miR-200a was determined by real-time PCR. A549 cells were transfected with Scr miR or Anti-miR followed by treatment with a CV or aPC. The expression of miR-200a **(C)** in the EVs or **(D)** EV-fused recipient BEAS-2B cells was analyzed by real-time PCR. ***P < .001; ****P < .0001; ns, not statistically significantly different.


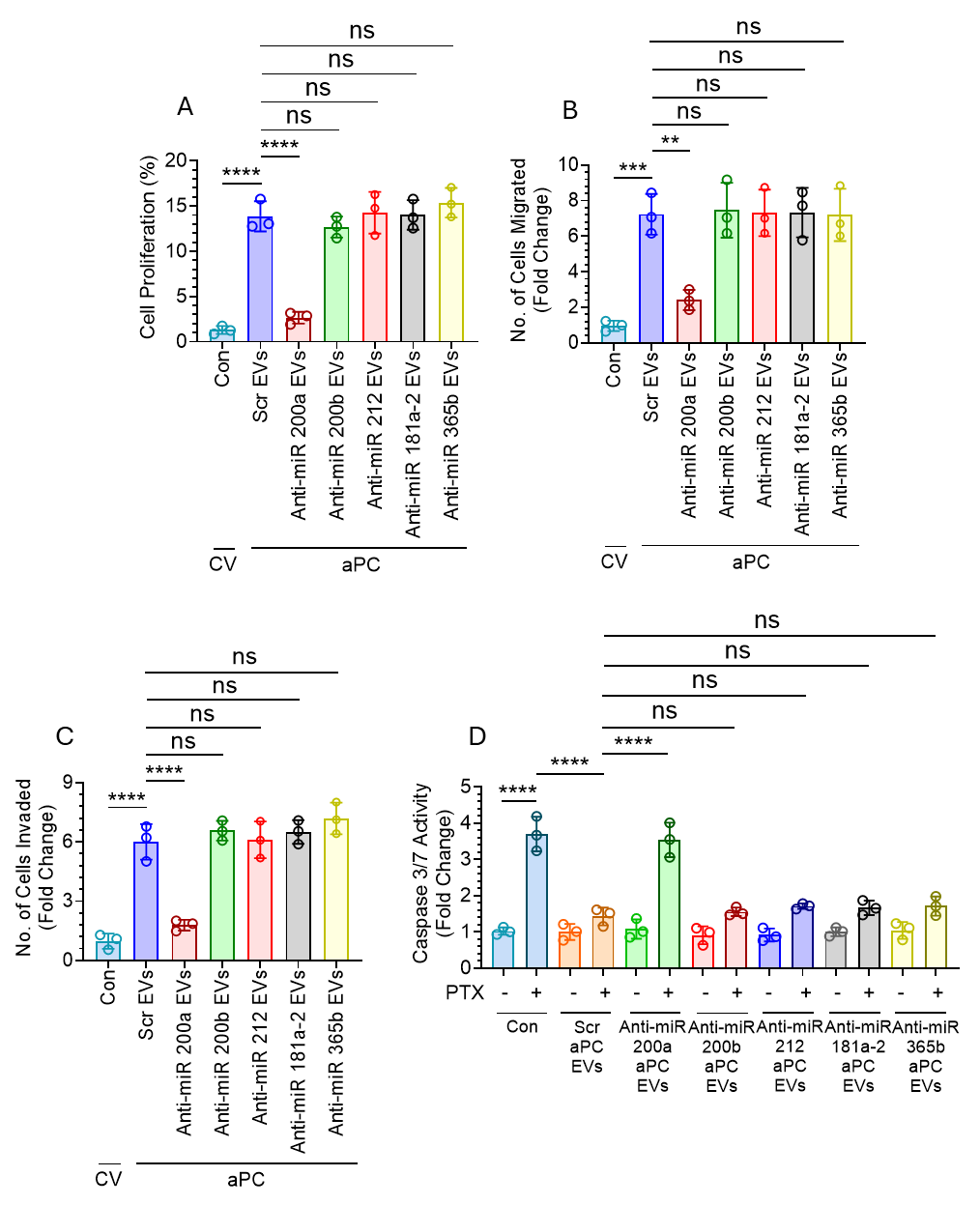
**Supplementary Fig. 5. aPC-EV-mediated enhanced proliferation, migration, invasion, and drug-induced apoptosis resistance was dependent on miR-200a rather than other upregulated miRs in the aPC-EVs, miR-200b, miR-212, miR-181a-2, and miR-365b.** A549 cells were transfected with 20 nM of Scr miR or Anti-miR 200a or Anti-miR 200b or Anti-miR 212 or Anti-miR 181a-2 or Anti-miR 365b for 48 h followed by treatment with aPC for another 16 h. EVs were isolated from the culture supernatant and quantified by NTA Nano-Sight. An equal number (1 X 10^8^) of EVs were fused with BEAS-2B cells for analyzing **(A)** proliferation, **(B)** migration, and **(C)** invasion. **(D)** The EVs were also fused with naïve A549 cells in equal number (1 X 10^8^) followed by PTX treatment for 48 h. Caspase 3/7 activity was measured to assess the degree of apoptosis. **P < .01; ***P < .001; ****P < .0001; ns, not statistically significantly different.

**Supplementary Fig. 6. Control EVs packaged with miR-200a mimics also promote cell proliferation, migration, and invasion as well as confer drug resistance. (A)** A549 cells were transfected with 20 nM of Scr miR or miR-200a mimic (miR Mimic). Forty-eight hours later, EVs were isolated from the culture supernatant and the expression of miR-200a in the EVs was analyzed by real time PCR. **(B)** An equal number (1 X 10^8^) of EVs isolated from Scr miR- or miR Mimic-transfected were fused with BEAS-2B cells and EV-fused BEAS-2B cells were subjected to analysis of miR-200a expression by real time PCR. EV-fused BEAS-2B cells were also analyzed for cell proliferation by **(C)** CFSE proliferation assay as well as **(D)** BrdU incorporation assay, **(E) and (F)** migration by trans-well migration assay, **(G) and (H)** invasion by Matrigel invasion assay in a trans-well chamber, and analysis of PTX-induced apoptosis by **(I)** Caspase3/7 activity assay as well as **(J) and (K)** Bax/Bcl-2 ratio analysis by western blotting. ***P < .001; ****P < .0001; ns, not statistically significantly different.

**
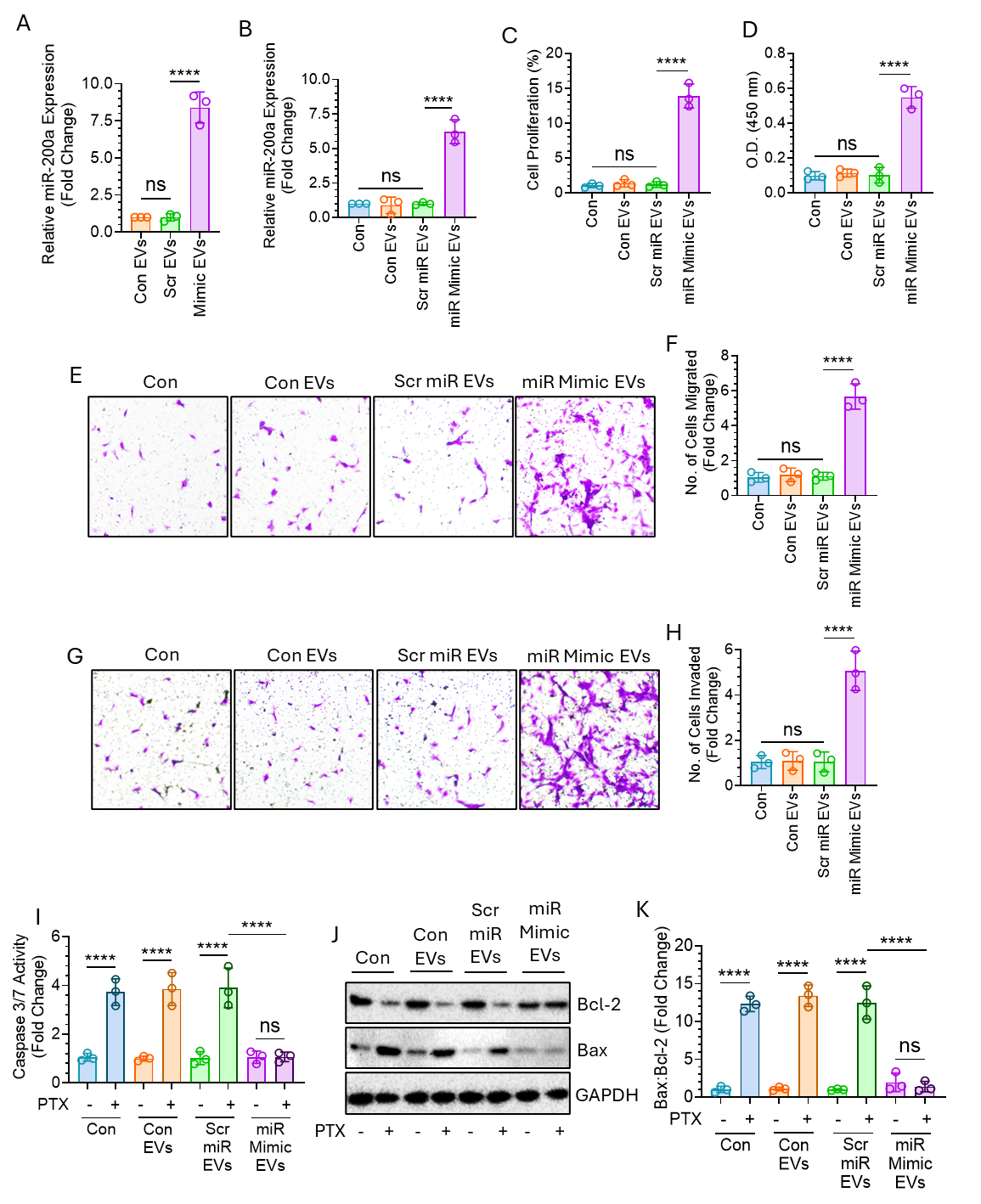
**


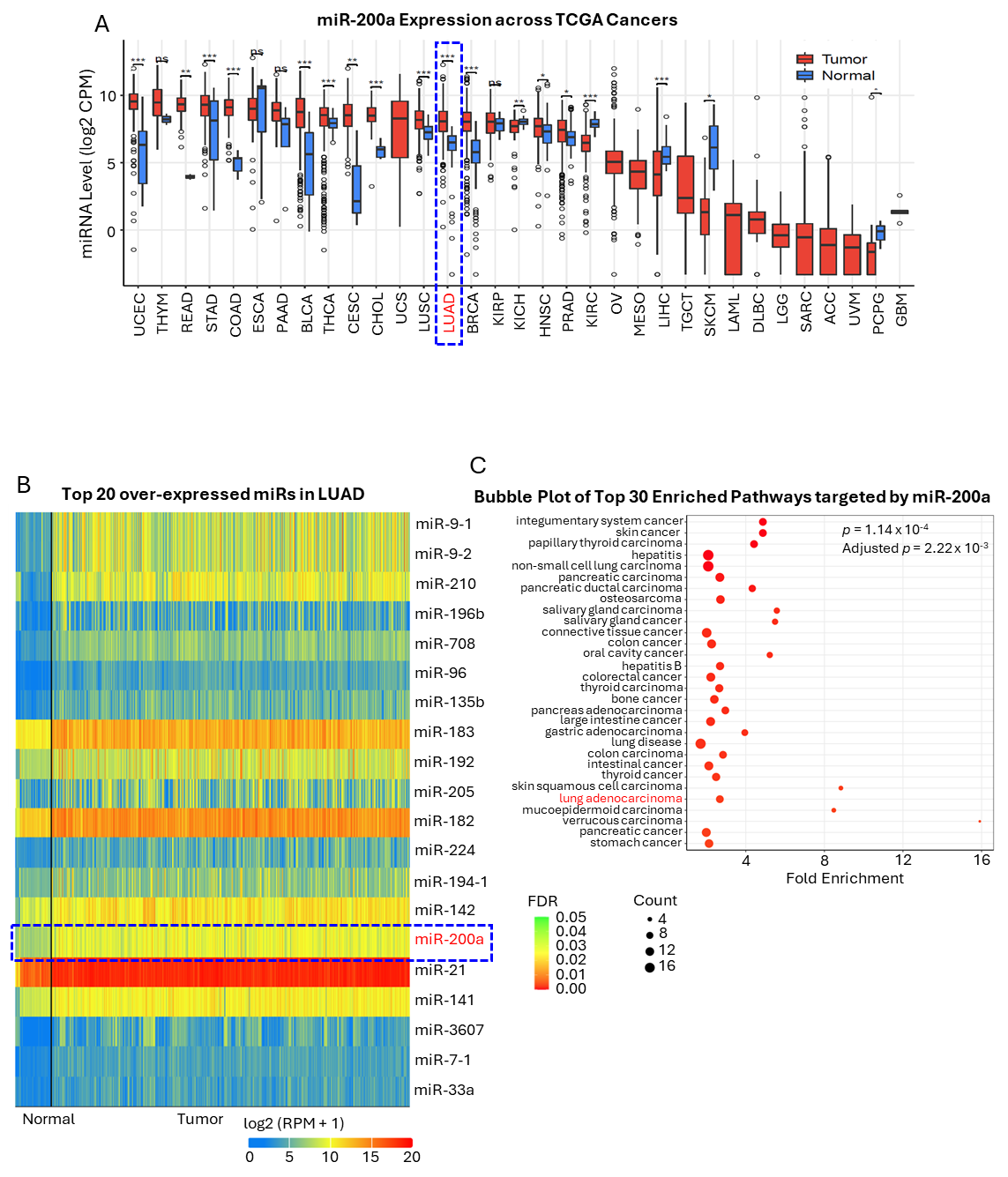
**Supplementary Fig. 7. miR-200a expression in LUAD samples as compared to healthy control. (A)** miR200a expression analysis using the Cancer MIRNome portal (<http://bioinfo.jialab-ucr.org/CancerMIRNome/>) in LUAD tumors as compared to normal tissues across multiple cancer types in the TCGA dataset as analyzed by Wilcoxon rank-sum test. **(B)** The top 20 overexpressed miRs in LUAD. **(C)** Bubble Plot of Top 30 Enriched Pathways targeted by miR-200a (p = 1.14 x 10^-4^). *P < .05; **P < .01; ***P < .001.


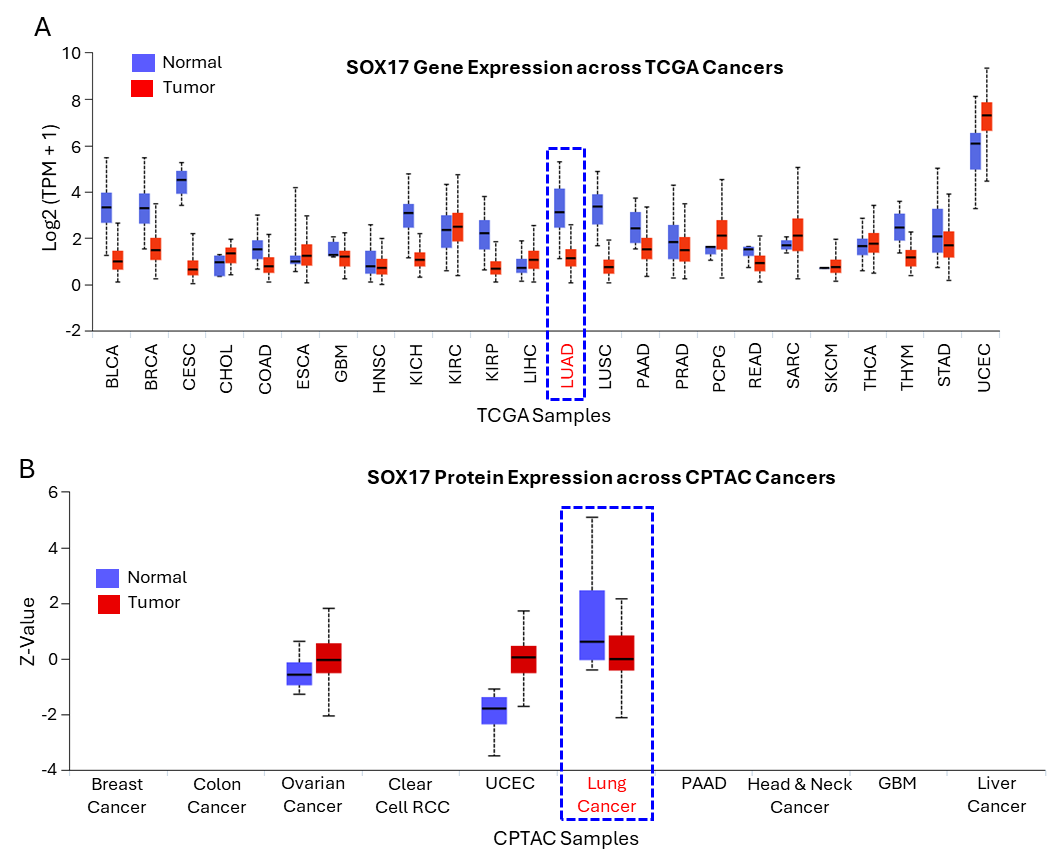
**Supplementary Fig. 8. SOX17 gene and protein expression in LUAD tissues as compared to normal by TCGA and CPTAC database, respectively. (A)** SOX17 gene expression analysis using the UALCAN portal in LUAD tumors compared to normal tissues across multiple cancer types in the TCGA dataset. **(B)** Protein expression analysis from the Clinical Proteomic Tumor Analysis Consortium (CPTAC) in LUAD tumors compared to normal tissues across multiple cancer types.

**
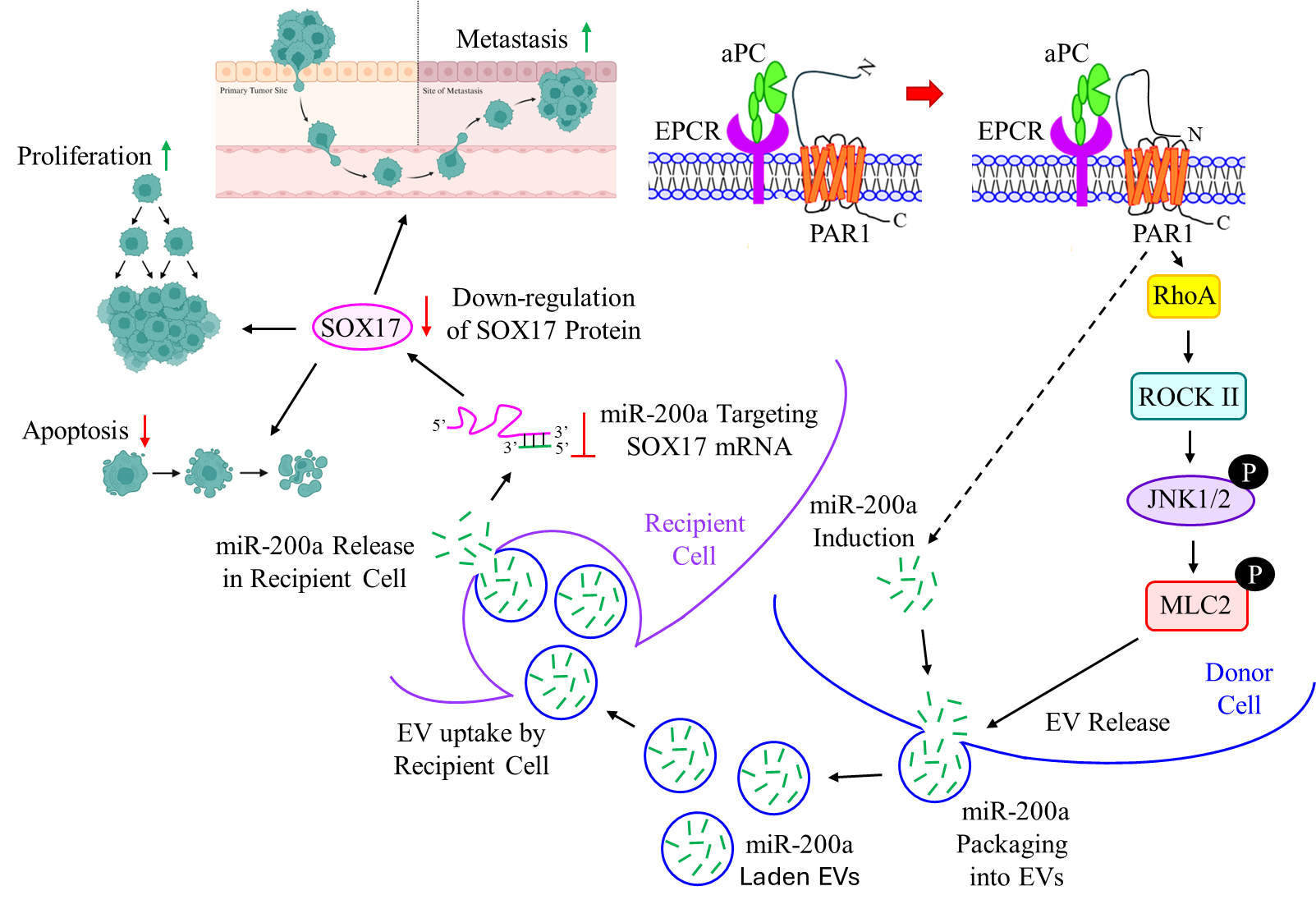
Supplementary Fig. 9. Schematic diagram showing that aPC-EPCR signaling promotes the release of miR-200a-laden EVs from A549 cells which induce proliferation, migration, invasion, and drug resistance via downregulating miR-200a target, SOX17.** aPC binds EPCR and cleaves PAR1 at the N-terminal end to generate a new N-termini which acts as a tethered ligand that binds to PAR1 itself and induces its activation. PAR1 activation leads to the activation of RhoA which further activates ROCKII. ROCKII activation further promotes JNK1/2 phosphorylation which in turn phosphorylates MLC2. Phospho-MLC2 plays a key role in the release of EVs. aPC-EPCR-PAR1 signaling also induces the expression of miR-200a which are readily packaged into aPC-derived EVs. EVs’ miR-200a are released into the cytosol of the recipient cells upon EV fusion where it targets SOX17 leading to the induction of metastasis, proliferation, and apoptosis resistance.
