# Supplementary material for: Activated protein C promotes human lung cancer progression through the release of tumor extracellular vesicles and transfer of microRNA-200a
Source: Cell Death Dis. 2025 Nov 21;16(1):848. doi: 10.1038/s41419-025-08173-z (PMC12638984; doi:10.1038/s41419-025-08173-z)
Supplement: Supplementary file 2 — Original Raw Data_Western blots and qRT-PCR [file 41419_2025_8173_MOESM2_ESM.docx]

**
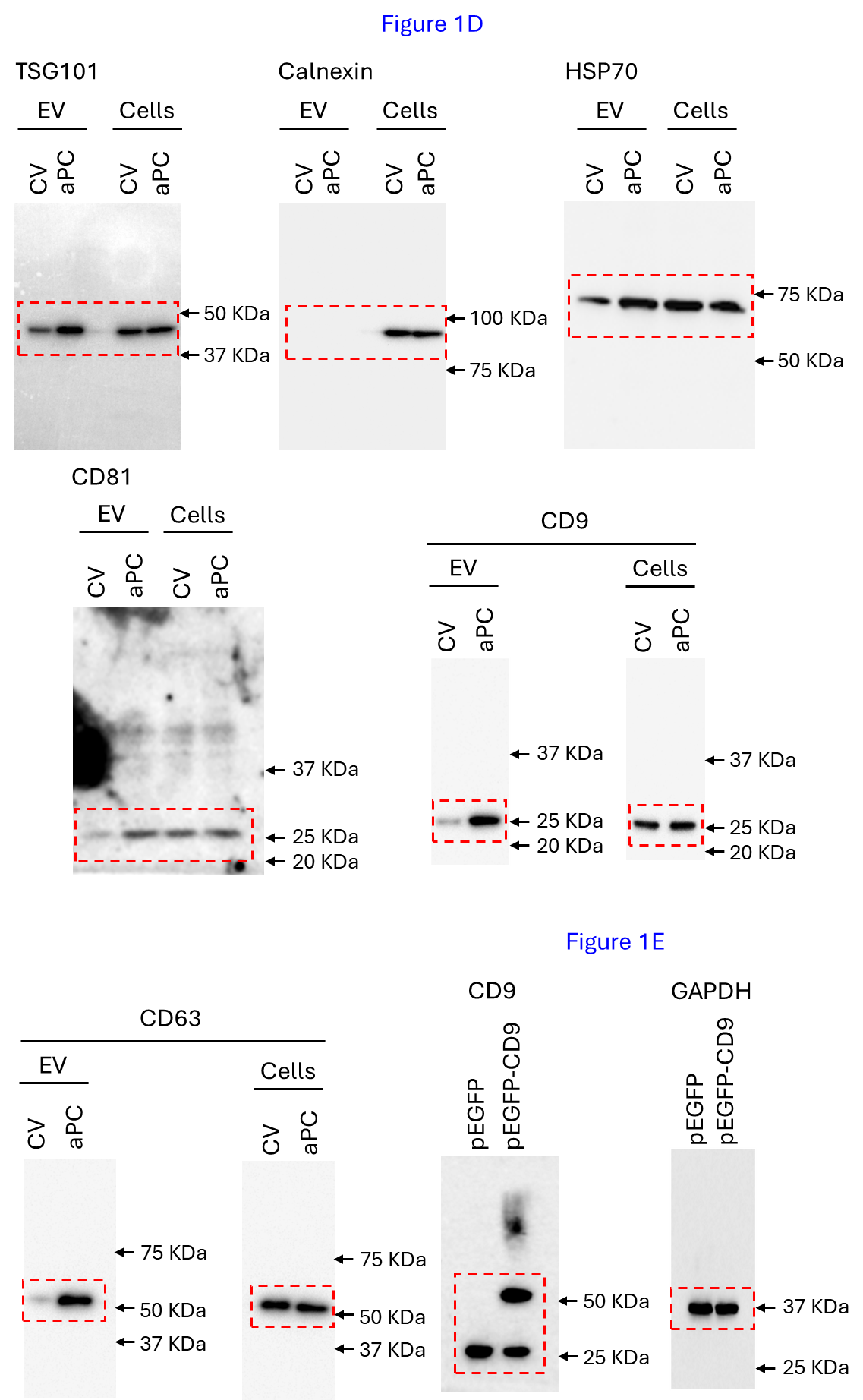
Original Western Blots**


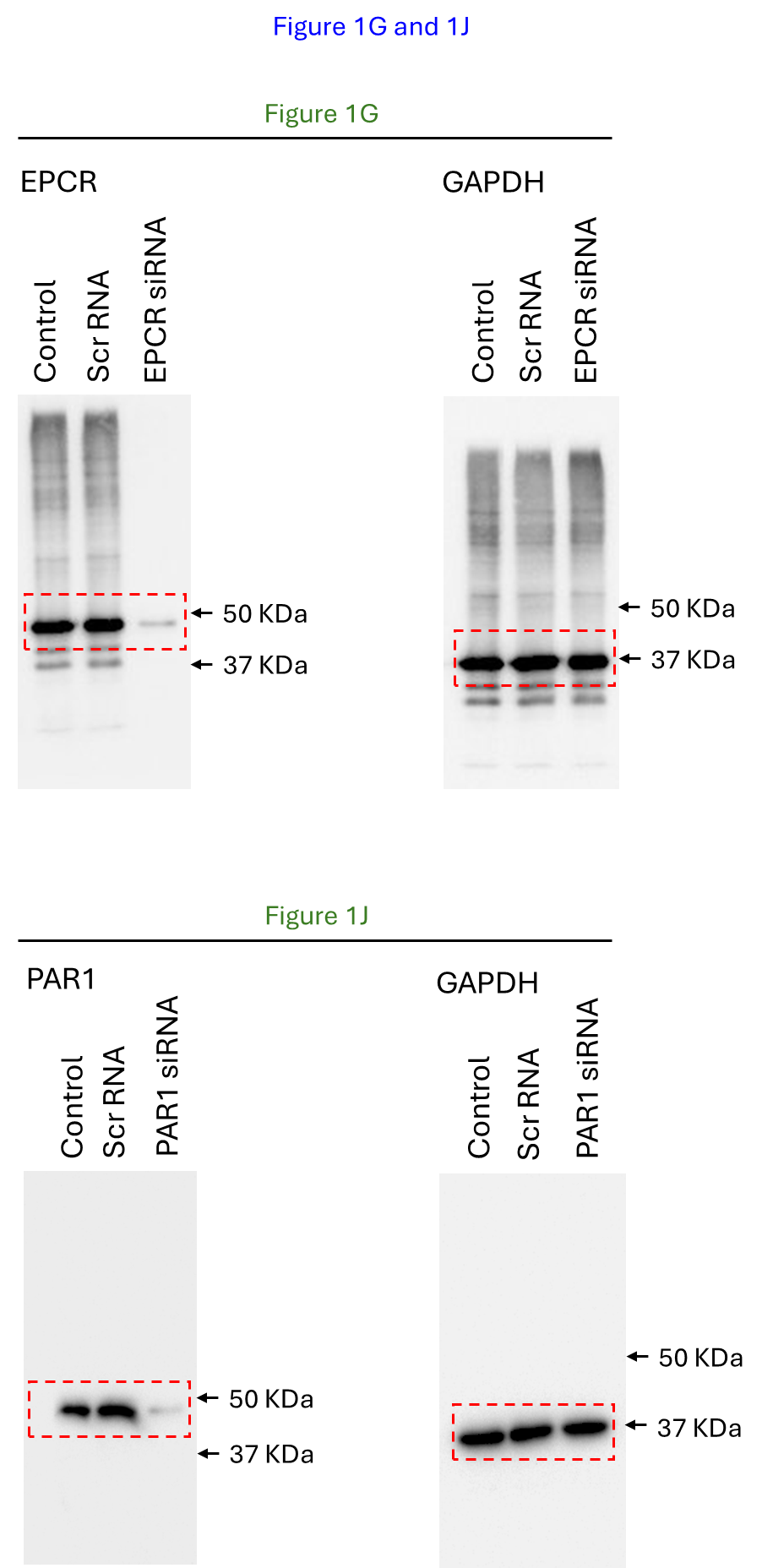


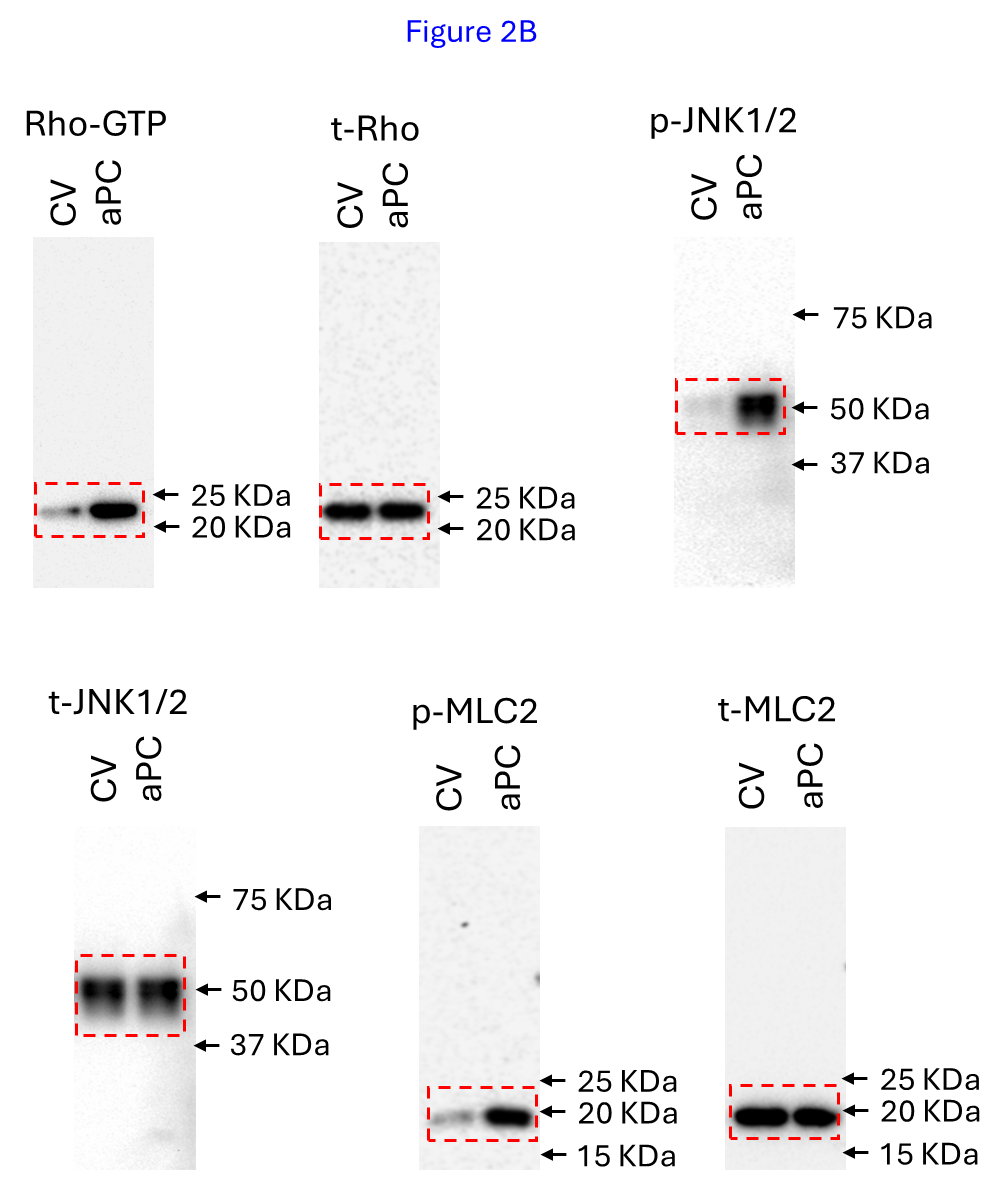


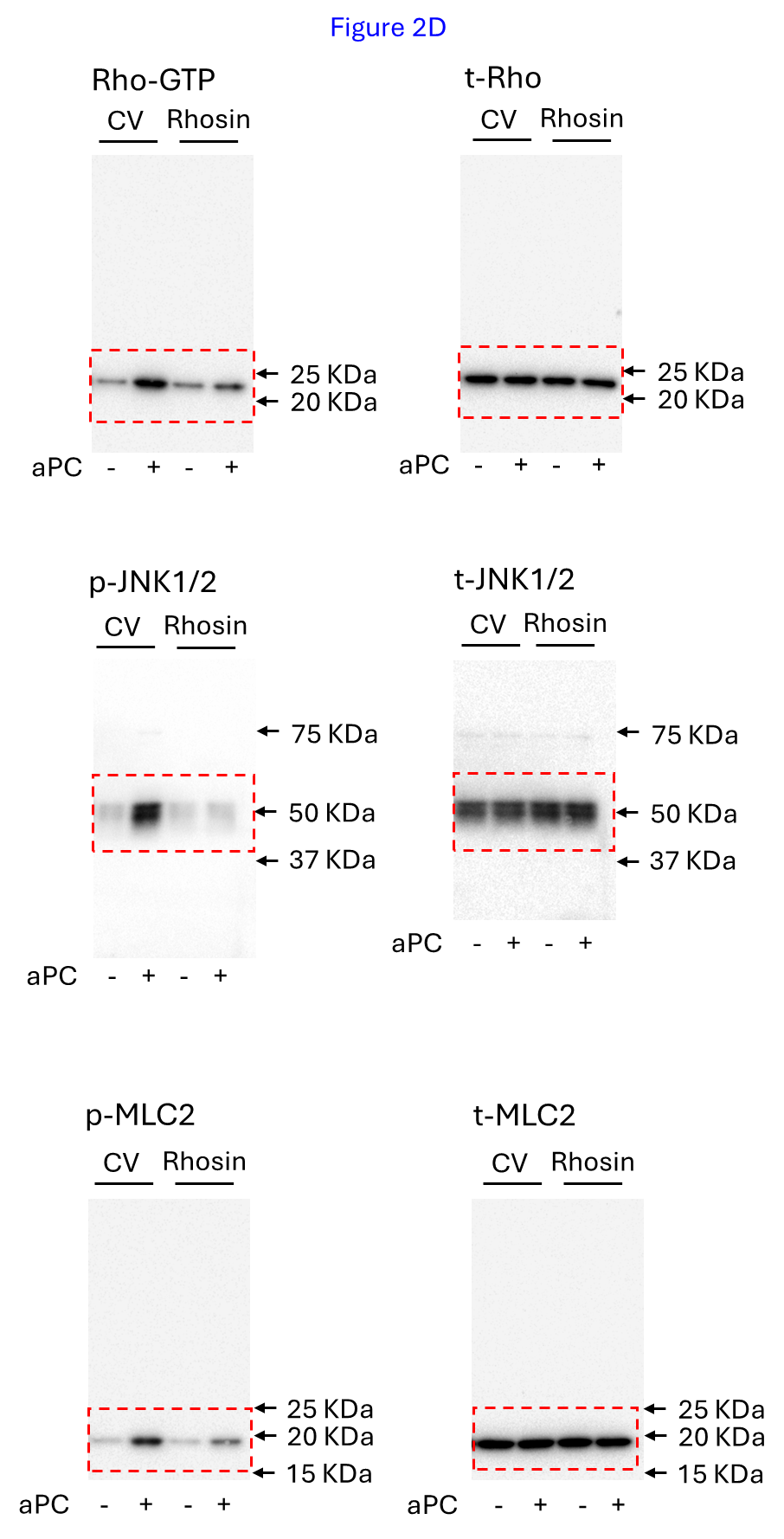


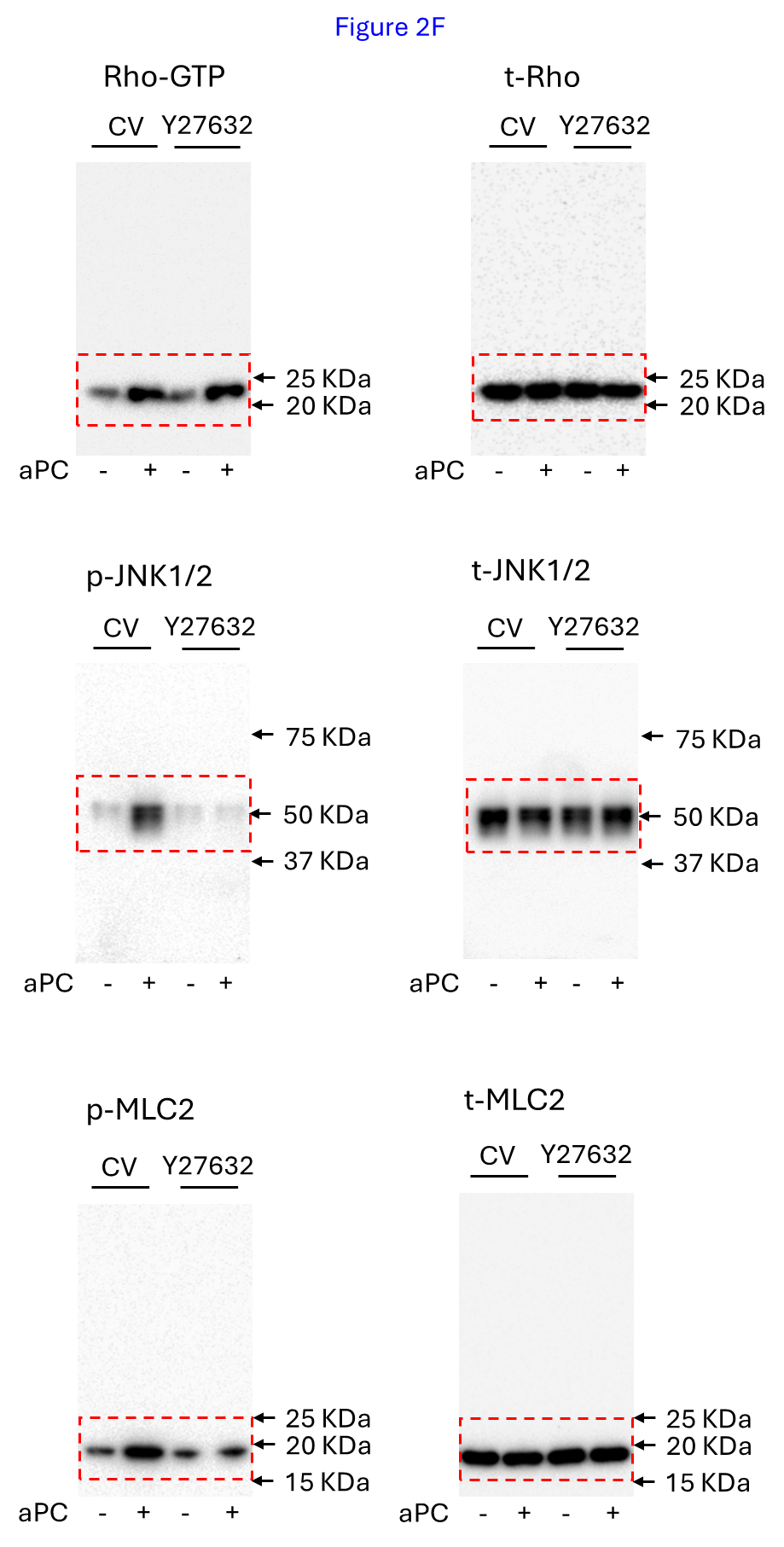


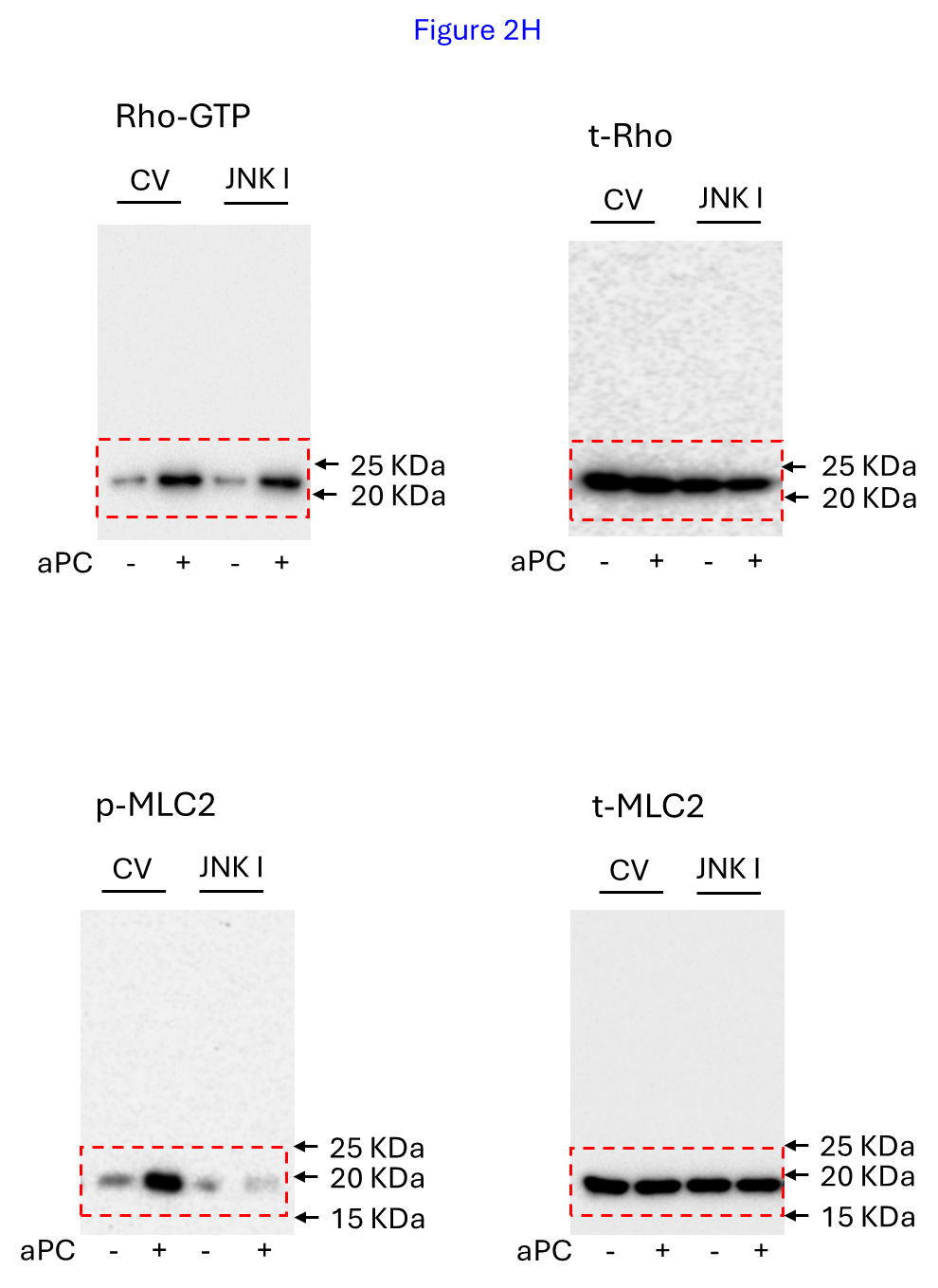


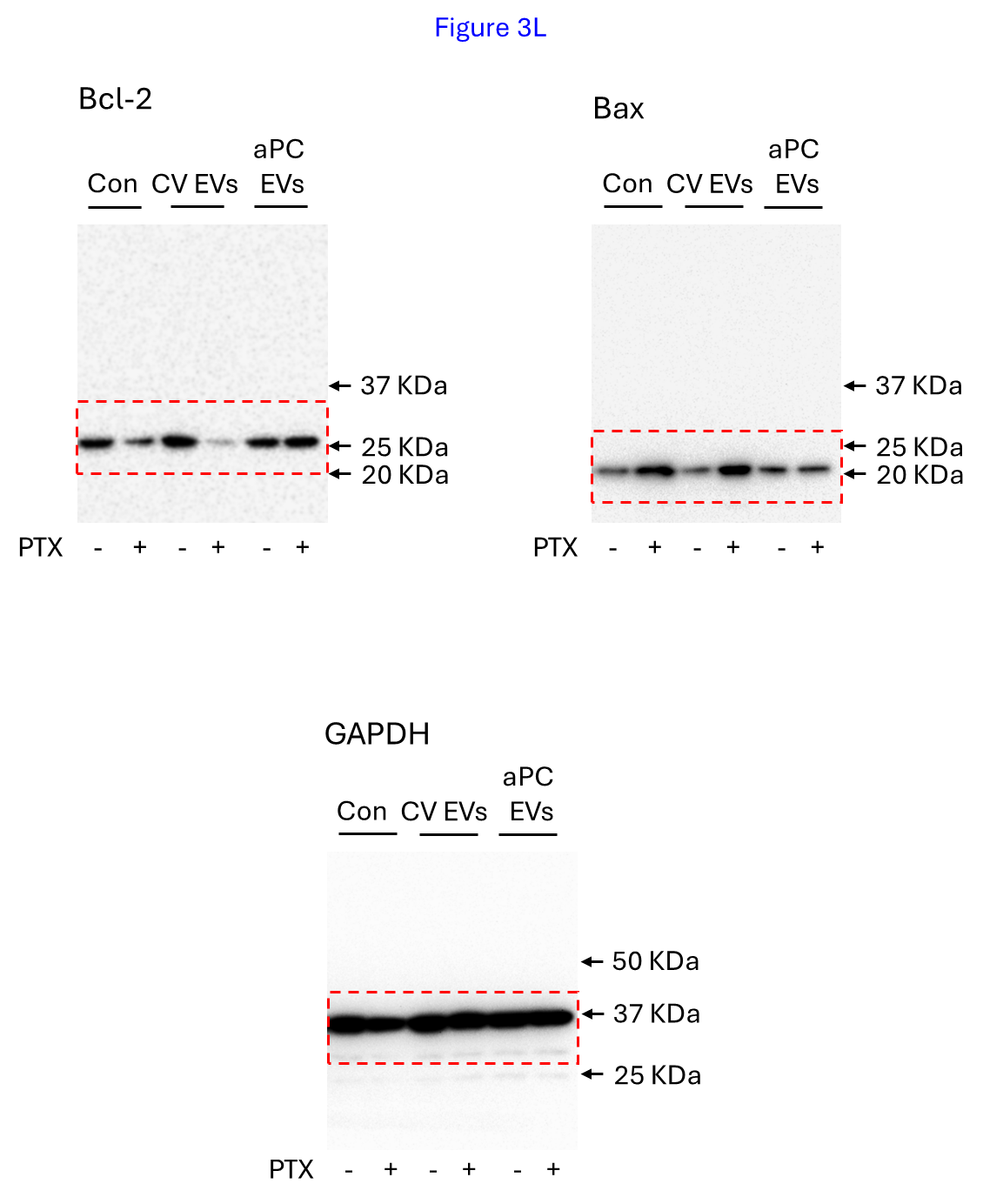


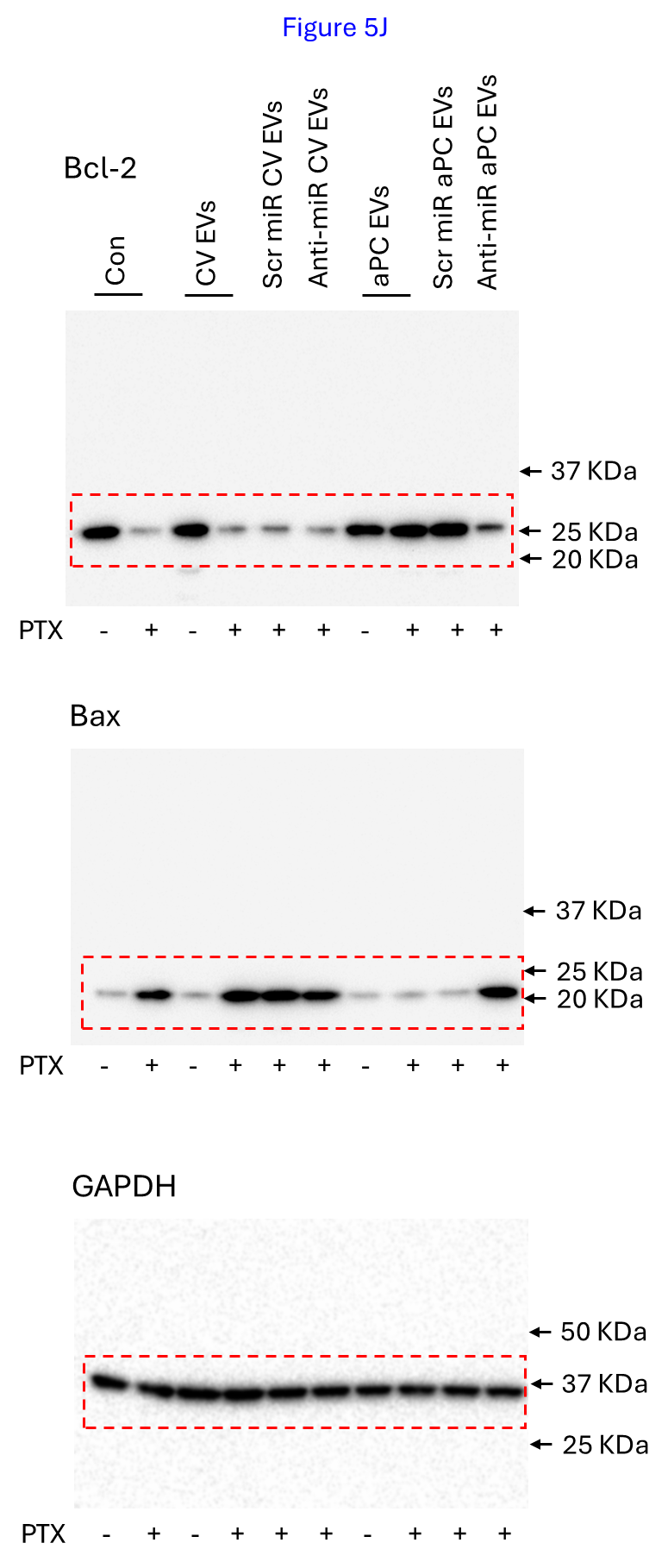


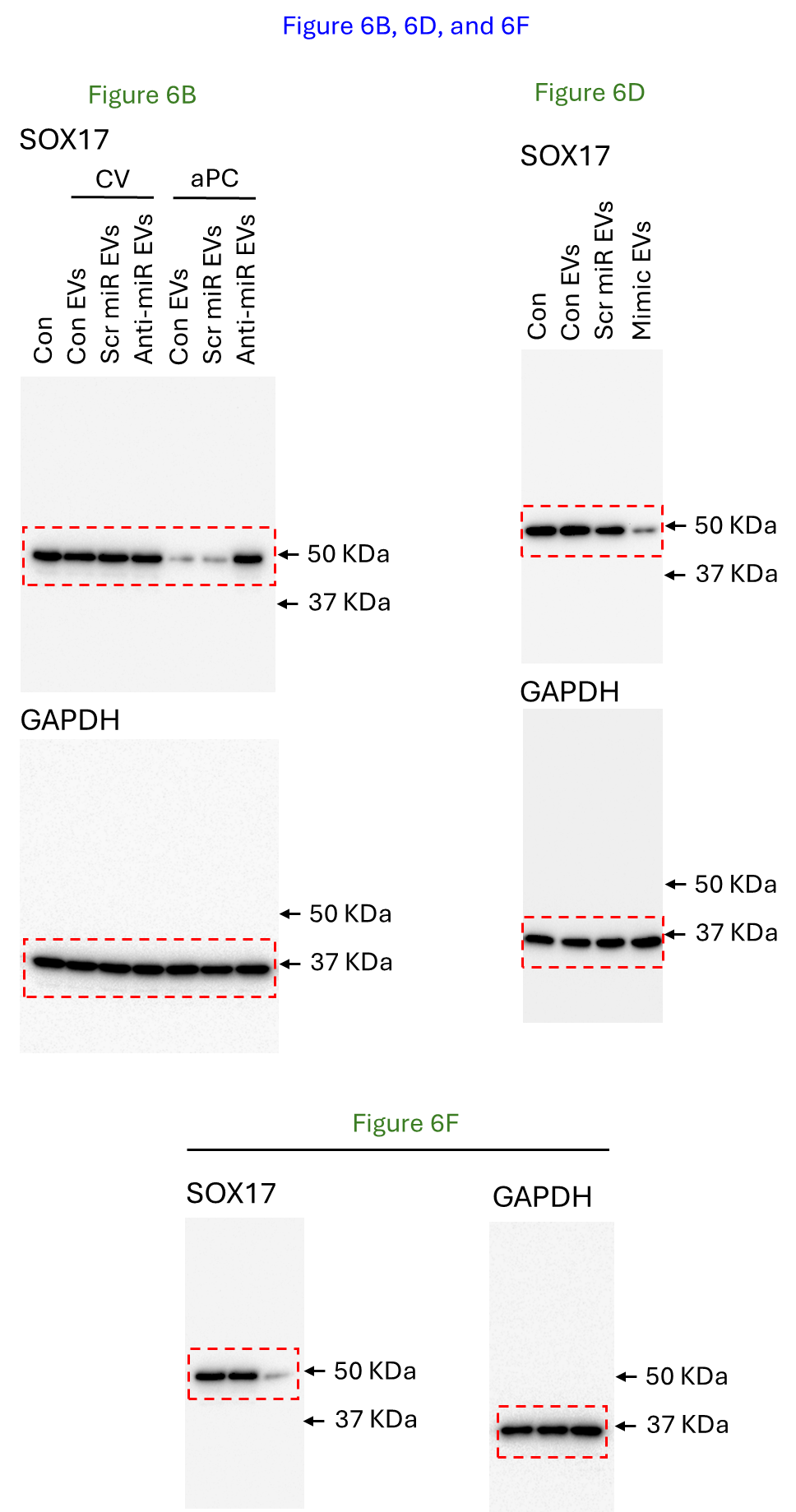


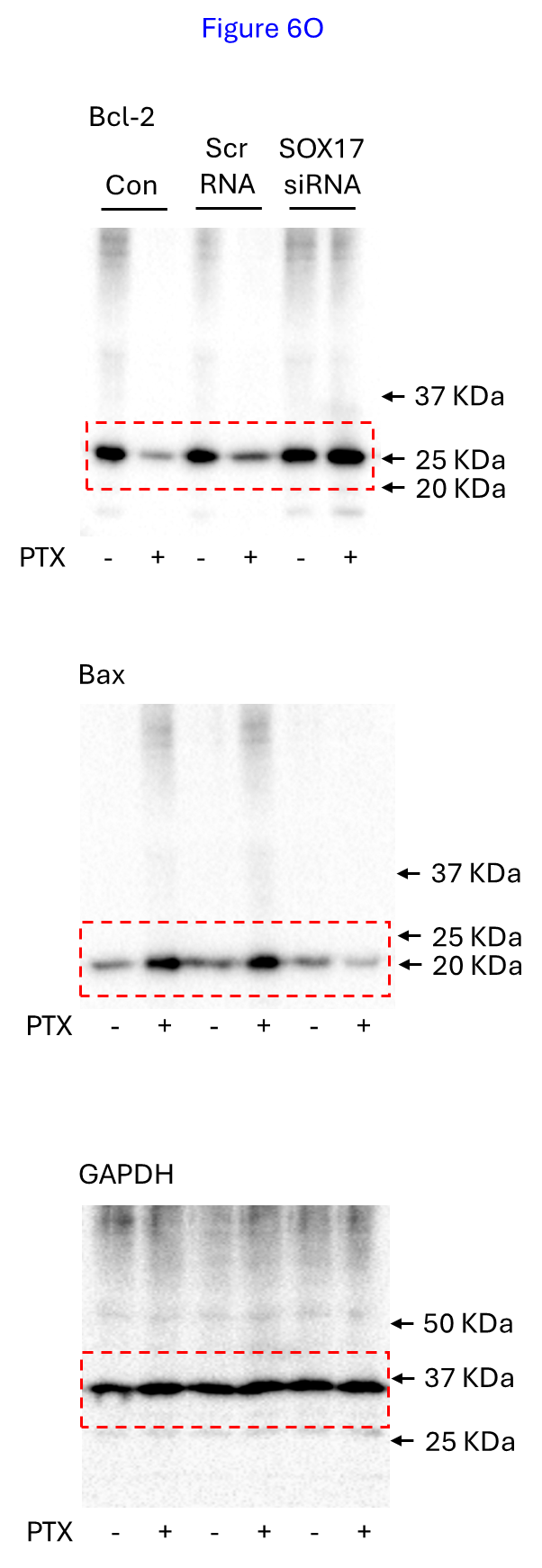


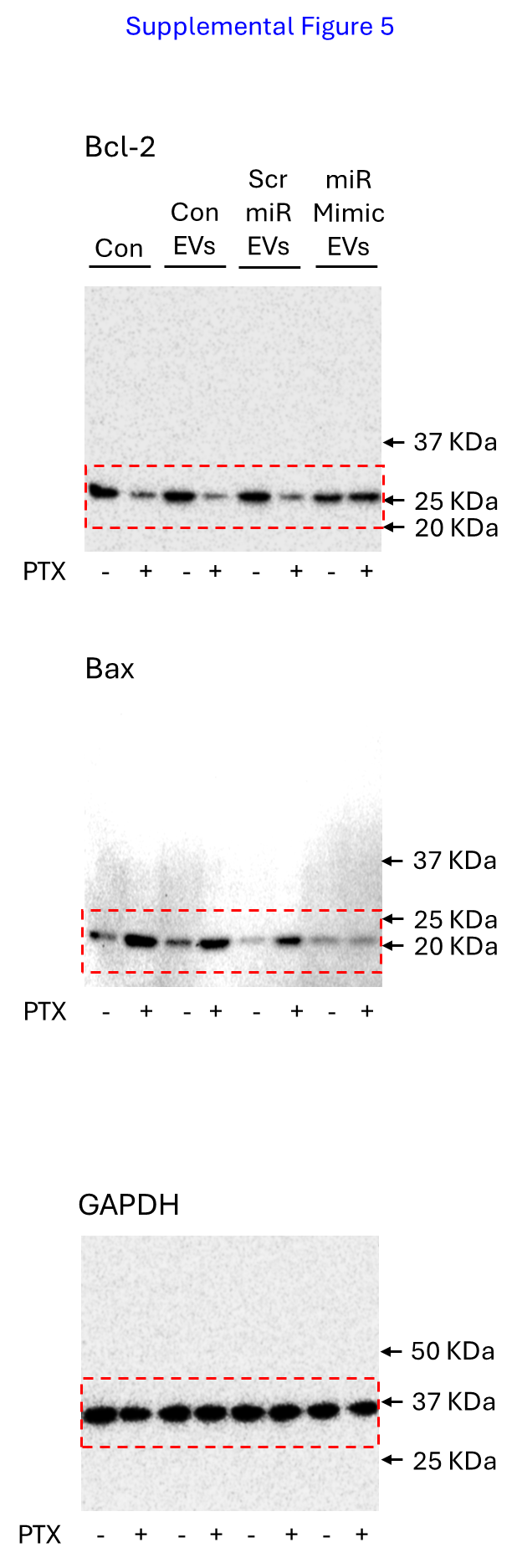


**Raw Data of Real-time PCR**

**Figure 4C**

| Set No. | Treatment | CT_Target_ | CT_Housekeeping_ | ∆CT (CT_Target_ - CT_Housekeeping_) | ∆∆CT (∆CT_Treatment_ -∆CT_Control_) | 2^-∆∆CT^ |
| --- | --- | --- | --- | --- | --- | --- |
| **1** | miR-181a-2_Con | 20.13 | 19.23 | 0.90 | 0 | 1 |
|  | miR-181a-2_aPC | 18.90 | 19.61 | -0.71 | -1.61 | 3.0525184179 |
|  | miR-200a_Con | 20.95 | 19.23 | 1.72 | 0.82 | 0.5664419426 |
|  | miR-200a_aPC | 17.86 | 19.61 | -1.75 | -2.65 | 6.2766727832 |
|  | miR-200b_Con | 21.53 | 19.23 | 2.30 | 1.40 | 0.3789291416 |
|  | miR-200b_aPC | 18.50 | 19.61 | -1.11 | -2.01 | 4.0278222002 |
|  | miR-365b_Con | 19.04 | 19.23 | -0.19 | -1.09 | 2.1287403649 |
|  | miR-365b_aPC | 17.95 | 19.61 | -1.66 | -2.56 | 5.8970768692 |
|  | miR-212_Con | 22.48 | 19.23 | 3.25 | 2.35 | 0.1961460245 |
|  | miR-212_aPC | 20.21 | 19.61 | 0.60 | -0.30 | 1.2311444133 |
|  | miR-615_Con | 18.26 | 19.23 | -0.97 | -1.87 | 3.6553258009 |
|  | miR-615_aPC | 20.07 | 19.61 | 0.46 | -0.44 | 1.3566043274 |
|  | miR-4521_Con | 19.46 | 19.23 | 0.23 | -0.67 | 1.5910729675 |
|  | miR-4521_aPC | 22.30 | 19.61 | 2.69 | 1.79 | 0.289172046 |
|  |  |  |  |  |  |  |
| **2** | miR-181a-2_Con | 20.19 | 21.12 | -0.93 | 0 | 1 |
|  | miR-181a-2_aPC | 19.05 | 21.82 | -2.77 | -1.84 | 3.5801002837 |
|  | miR-200a_Con | 20.62 | 21.12 | -0.50 | 0.43 | 0.7422617853 |
|  | miR-200a_aPC | 18.36 | 21.82 | -3.46 | -2.53 | 5.7757167821 |
|  | miR-200b_Con | 21.09 | 21.12 | -0.03 | 0.90 | 0.5358867313 |
|  | miR-200b_aPC | 18.72 | 21.82 | -3.10 | -2.17 | 4.5002339388 |
|  | miR-365b_Con | 18.87 | 21.12 | -2.25 | -1.32 | 2.4966610978 |
|  | miR-365b_aPC | 18.54 | 21.82 | -3.28 | -2.35 | 5.0982425093 |
|  | miR-212_Con | 20.50 | 21.12 | 0.62 | 1.55 | 0.3415100642 |
|  | miR-212_aPC | 20.87 | 21.82 | -0.95 | -0.02 | 1.0139594798 |
|  | miR-615_Con | 18.06 | 21.12 | -3.06 | -2.13 | 4.377174805 |
|  | miR-615_aPC | 20.74 | 21.82 | -1.08 | -0.15 | 1.1095694721 |
|  | miR-4521_Con | 19.73 | 21.12 | -1.39 | -0.46 | 1.3755418181 |
|  | miR-4521_aPC | 22.24 | 21.82 | 0.42 | 1.35 | 0.3922920489 |
|  |  |  |  |  |  |  |
| **3** | miR-181a-2_Con | 18.29 | 17.09 | 1.20 | 0 | 1 |
|  | miR-181a-2_aPC | 17.20 | 17.52 | -0.32 | -1.52 | 2.867910496 |
|  | miR-200a_Con | 19.79 | 17.09 | 2.70 | 1.50 | 0.3535533906 |
|  | miR-200a_aPC | 16.02 | 17.52 | -1.50 | -2.70 | 6.4980191708 |
|  | miR-200b_Con | 18.75 | 17.09 | 1.66 | 0.46 | 0.7269862587 |
|  | miR-200b_aPC | 16.80 | 17.52 | -0.72 | -1.92 | 3.7842305869 |
|  | miR-365b_Con | 17.31 | 17.09 | 0.22 | -0.98 | 1.972465409 |
|  | miR-365b_aPC | 16.44 | 17.52 | -1.08 | -2.28 | 4.8567795376 |
|  | miR-212_Con | 20.09 | 17.09 | 3.00 | 1.80 | 0.2871745887 |
|  | miR-212_aPC | 19.06 | 17.52 | 1.54 | 0.34 | 0.7900413119 |
|  | miR-615_Con | 16.09 | 17.09 | -1.00 | -2.20 | 4.59479342 |
|  | miR-615_aPC | 18.07 | 17.52 | 0.55 | -0.65 | 1.5691681958 |
|  | miR-4521_Con | 17.51 | 17.09 | 0.42 | -0.78 | 1.7171308729 |
|  | miR-4521_aPC | 19.82 | 17.52 | 2.30 | 1.10 | 0.4665164958 |

**Figure 5A**

| Set No. | Treatment | CT_Target_ | CT_Housekeeping_ | ∆CT (CT_Target_ - CT_Housekeeping_) | ∆∆CT (∆CT_Treatment_ -∆CT_Control_) | 2^-∆∆CT^ |
| --- | --- | --- | --- | --- | --- | --- |
| **1** | Con | 21.98 | 20.17 | 1.81 | 0 | 1 |
|  | Con EVs | 21.90 | 20.87 | 1.03 | -0.78 | 1.7171308729 |
|  | aPC EVs | 19.31 | 20.53 | -1.22 | -3.03 | 8.1680970057 |
|  |  |  |  |  |  |  |
| **2** | Con | 18.28 | 19.12 | -0.84 | 0 | 1 |
|  | Con EVs | 18.31 | 19.84 | -1.53 | -0.69 | 1.6132835184 |
|  | aPC EVs | 15.63 | 19.37 | -3.74 | -2.90 | 7.4642639323 |
|  |  |  |  |  |  |  |
| **3** | Con | 20.13 | 18.24 | 1.89 | 0 | 1 |
|  | Con EVs | 19.98 | 18.71 | 1.27 | -0.62 | 1.5368751813 |
|  | aPC EVs | 17.14 | 18.05 | -0.91 | -2.80 | 6.9644045064 |

**Figure 5B**

| Set No. | Treatment | CT_Target_ | CT_Housekeeping_ | ∆CT (CT_Target_ - CT_Housekeeping_) | ∆∆CT (∆CT_Treatment_ -∆CT_Control_) | 2^-∆∆CT^ |
| --- | --- | --- | --- | --- | --- | --- |
| **1** | Con | 20.15 | 19.31 | 0.84 | 0 | 1 |
|  | Actn D | 20.05 | 19.48 | 0.57 | -0.27 | 1.2058078277 |
|  | CV EVs_Con | 19.13 | 19.01 | 0.12 | -0.72 | 1.6471820345 |
|  | CV EVs_Actn D | 19.40 | 19.73 | -0.33 | -1.17 | 2.2501169694 |
|  | aPC EVs_Con | 17.38 | 19.91 | -2.53 | -3.37 | 10.338822645 |
|  | aPC EVs_Actn D | 17.49 | 19.51 | -2.02 | -2.86 | 7.2601532425 |
|  |  |  |  |  |  |  |
| **2** | Con | 18.12 | 17.02 | 1.10 | 0 | 1 |
|  | Actn D | 17.94 | 17.24 | 0.70 | -0.40 | 1.3195079108 |
|  | CV EVs_Con | 18.41 | 17.91 | 0.50 | -0.60 | 1.5157165665 |
|  | CV EVs_Actn D | 17.82 | 17.55 | 0.27 | -0.83 | 1.7776853623 |
|  | aPC EVs_Con | 15.15 | 17.08 | -1.93 | -3.03 | 8.1680970057 |
|  | aPC EVs_Actn D | 15.79 | 17.82 | -2.03 | -3.13 | 8.7543496101 |
|  |  |  |  |  |  |  |
| **3** | Con | 19.12 | 21.37 | -2.25 | 0 | 1 |
|  | Actn D | 19.08 | 21.91 | -2.83 | -0.58 | 1.4948492486 |
|  | CV EVs_Con | 18.77 | 21.43 | -2.66 | -0.41 | 1.3286858141 |
|  | CV EVs_Actn D | 18.35 | 21.08 | -2.73 | -0.48 | 1.3947436664 |
|  | aPC EVs_Con | 16.32 | 21.45 | -5.13 | -2.88 | 7.361501205 |
|  | aPC EVs_Actn D | 16.62 | 21.86 | -5.24 | -2.99 | 7.9447399635 |

**Supplemental Figure 3A**

| Set No. | Treatment | CT_Target_ | CT_Housekeeping_ | ∆CT (CT_Target_ - CT_Housekeeping_) | ∆∆CT (∆CT_Treatment_ -∆CT_Control_) | 2^-∆∆CT^ |
| --- | --- | --- | --- | --- | --- | --- |
| **1** | CV | 21.02 | 17.74 | 3.28 | 0 | 1 |
|  | Actn D | 25.54 | 17.25 | 8.29 | 5.01 | 0.03103414 |
|  |  |  |  |  |  |  |
| **2** | CV | 21.61 | 17.1 | 4.51 | 0 | 1 |
|  | Actn D | 24.54 | 18.25 | 6.29 | 1.78 | 0.291183397 |
|  |  |  |  |  |  |  |
| **3** | CV | 22.25 | 20.02 | 2.23 | 0 | 1 |
|  | Actn D | 25.5 | 20.57 | 4.93 | 2.7 | 0.153893052 |

**Supplemental Figure 3B**

| Set No. | Treatment | CT_Target_ | CT_Housekeeping_ | ∆CT (CT_Target_ - CT_Housekeeping_) | ∆∆CT (∆CT_Treatment_ -∆CT_Control_) | 2^-∆∆CT^ |
| --- | --- | --- | --- | --- | --- | --- |
| **1** | CV | 16.60 | 14.25 | 2.35 | 0 | 1 |
|  | aPC | 18.54 | 17.02 | -1.52 | -3.87 | 14.6213032 |
|  |  |  |  |  |  |  |
| **2** | CV | 15.59 | 16.94 | 1.35 | 0 | 1 |
|  | aPC | 19.56 | 17.57 | -1.99 | -3.34 | 10.12605275 |
|  |  |  |  |  |  |  |
| **3** | CV | 15.02 | 17.77 | 2.75 | 0 | 1 |
|  | aPC | 18.88 | 18.43 | -0.45 | -3.2 | 9.18958684 |

**Supplemental Figure 3C**

| Set No. | Treatment | CT_Target_ | CT_Housekeeping_ | ∆CT (CT_Target_ - CT_Housekeeping_) | ∆∆CT (∆CT_Treatment_ -∆CT_Control_) | 2^-∆∆CT^ |
| --- | --- | --- | --- | --- | --- | --- |
| **1** | CV_Con EVs | 21.1 | 20.25 | 0.85 | 0 | 1 |
|  | CV_Scr miR EVs | 21 | 20.1 | 0.9 | 0.05 | 0.965936329 |
|  | CV_Anti-miR EVs | 20.69 | 19.26 | 1.43 | 0.53 | 0.692554734 |
|  | aPC_Con EVs | 18.79 | 21.02 | -2.23 | -3.08 | 8.456144324 |
|  | aPC_Scr miR EVs | 18.75 | 21.11 | -2.36 | -3.21 | 9.253505471 |
|  | aPC_Anti-miR EVs | 22.02 | 20.57 | 1.45 | 0.6 | 0.659753955 |
|  |  |  |  |  |  |  |
| **2** | CV_Con EVs | 20.75 | 19.08 | 1.67 | 0 | 1 |
|  | CV_Scr miR EVs | 21.79 | 20.19 | 1.60 | -0.07 | 1.0497166836 |
|  | CV_Anti-miR EVs | 19.83 | 18.29 | 1.54 | -0.13 | 1.0942937013 |
|  | aPC_Con EVs | 17.31 | 18.82 | -1.51 | -3.18 | 9.0630710824 |
|  | aPC_Scr miR EVs | 17.93 | 19.17 | -1.24 | -2.91 | 7.5161819937 |
|  | aPC_Anti-miR EVs | 21.29 | 19.93 | 1.36 | -0.31 | 1.2397076999 |
|  |  |  |  |  |  |  |
| **3** | CV_Con EVs | 19.73 | 18.25 | 1.48 | 0 | 1 |
|  | CV_Scr miR EVs | 20.20 | 19.17 | 1.03 | -0.45 | 1.3660402568 |
|  | CV_Anti-miR EVs | 20.47 | 18.72 | 1.75 | 0.27 | 0.8293195458 |
|  | aPC_Con EVs | 17.31 | 19.02 | -1.71 | -3.19 | 9.1261097269 |
|  | aPC_Scr miR EVs | 17.26 | 18.76 | -1.50 | -2.98 | 7.8898616359 |
|  | aPC_Anti-miR EVs | 20.58 | 19.01 | 1.57 | 0.09 | 0.9395227492 |

**Supplemental Figure 3D**

| Set No. | Treatment | CT_Target_ | CT_Housekeeping_ | ∆CT (CT_Target_ - CT_Housekeeping_) | ∆∆CT (∆CT_Treatment_ -∆CT_Control_) | 2^-∆∆CT^ |
| --- | --- | --- | --- | --- | --- | --- |
|  | Con | 20.78 | 18.53 | 2.25 | 0 | 1 |
| **1** | CV_Con EVs | 19.91 | 18.05 | 1.86 | -0.39 | 1.3103934039 |
|  | CV_Scr miR EVs | 20.45 | 18.89 | 1.56 | -0.69 | 1.6132835184 |
|  | CV_Anti-miR EVs | 21.91 | 19.09 | 2.82 | 0.57 | 0.6736167884 |
|  | aPC_Con EVs | 18.16 | 18.76 | -0.60 | -2.85 | 7.2100037009 |
|  | aPC_Scr miR EVs | 18.05 | 18.55 | -0.50 | -2.75 | 6.727171322 |
|  | aPC_Anti-miR EVs | 20.36 | 18.23 | 2.13 | -0.12 | 1.0867348625 |
|  |  |  |  |  |  |  |
| **2** | Con | 17.12 | 18.01 | -0.89 | 0 | 1 |
|  | CV_Con EVs | 17.34 | 18.37 | -1.03 | -0.14 | 1.1019051159 |
|  | CV_Scr miR EVs | 17.86 | 18.98 | -1.12 | -0.23 | 1.1728349492 |
|  | CV_Anti-miR EVs | 17.87 | 18.35 | -0.48 | 0.41 | 0.7526233737 |
|  | aPC_Con EVs | 15.21 | 18.61 | -3.40 | -2.51 | 5.6962007824 |
|  | aPC_Scr miR EVs | 14.98 | 18.12 | -3.14 | -2.25 | 4.75682846 |
|  | aPC_Anti-miR EVs | 17.96 | 18.76 | -0.80 | 0.09 | 0.9395227492 |
|  |  |  |  |  |  |  |
| **3** | Con | 19.37 | 21.25 | -1.88 | 0 | 1 |
|  | CV_Con EVs | 19.33 | 21.03 | -1.70 | 0.18 | 0.8827029963 |
|  | CV_Scr miR EVs | 20.39 | 22.01 | -1.62 | 0.26 | 0.8350879194 |
|  | CV_Anti-miR EVs | 19.97 | 21.06 | -1.09 | 0.79 | 0.578344092 |
|  | aPC_Con EVs | 17.26 | 21.83 | -4.57 | -2.69 | 6.4531340738 |
|  | aPC_Scr miR EVs | 16.36 | 21.07 | -4.71 | -2.83 | 7.1107414493 |
|  | aPC_Anti-miR EVs | 18.14 | 20.37 | -2.23 | -0.35 | 1.2745606273 |

**Supplemental Figure 5A**

| Set No. | Treatment | CT_Target_ | CT_Housekeeping_ | ∆CT (CT_Target_ - CT_Housekeeping_) | ∆∆CT (∆CT_Treatment_ -∆CT_Control_) | 2^-∆∆CT^ |
| --- | --- | --- | --- | --- | --- | --- |
| **1** | Con EVs | 18.31 | 16.18 | 2.13 | 0 | 1 |
|  | Scr miR EVs | 19.42 | 16.84 | 2.58 | 0.45 | 0.732042848 |
|  | miR Mimic EVs | 15.52 | 16.53 | -1.01 | -3.14 | 8.815240927 |
|  |  |  |  |  |  |  |
| **2** | Con EVs | 19.31 | 20.31 | -1.00 | 0 | 1 |
|  | Scr miR EVs | 19.86 | 20.97 | -1.11 | -0.11 | 1.0792282365 |
|  | miR Mimic EVs | 16.26 | 20.46 | -4.20 | -3.20 | 9.18958684 |
|  |  |  |  |  |  |  |
| **3** | Con EVs | 17.81 | 18.18 | -0.37 | 0 | 1 |
|  | Scr miR EVs | 17.89 | 18.51 | -0.62 | -0.25 | 1.189207115 |
|  | miR Mimic EVs | 15.41 | 18.63 | -3.22 | -2.85 | 7.2100037009 |

**Supplemental Figure 5B**

| Set No. | Treatment | CT_Target_ | CT_Housekeeping_ | ∆CT (CT_Target_ - CT_Housekeeping_) | ∆∆CT (∆CT_Treatment_ -∆CT_Control_) | 2^-∆∆CT^ |
| --- | --- | --- | --- | --- | --- | --- |
| **1** | Con | 19.25 | 18.37 | 0.88 | 0 | 1 |
|  | Con EVs | 19.28 | 18.79 | 0.49 | -0.39 | 1.3103934039 |
|  | Scr miR EVs | 19.11 | 18.21 | 0.90 | 0.02 | 0.9862327045 |
|  | miR Mimic EVs | 16.88 | 18.59 | -1.71 | -2.59 | 6.0209869896 |
|  |  |  |  |  |  |  |
| **2** | Con | 18.23 | 17.29 | 0.94 | 0 | 1 |
|  | Con EVs | 18.59 | 17.83 | 0.76 | -0.18 | 1.1328838853 |
|  | Scr miR EVs | 18.70 | 17.61 | 1.09 | 0.15 | 0.9012504626 |
|  | miR Mimic EVs | 15.51 | 17.02 | -1.51 | -2.45 | 5.464161027 |
|  |  |  |  |  |  |  |
| **3** | Con | 18.34 | 21.09 | -2.75 | 0 | 1 |
|  | Con EVs | 21.33 | 21.79 | -0.46 | 2.29 | 0.2044755146 |
|  | Scr miR EVs | 18.64 | 21.54 | -2.90 | -0.15 | 1.1095694721 |
|  | miR Mimic EVs | 15.74 | 21.33 | -5.59 | -2.84 | 7.1602005674 |
